# Accurate Construction of Photoactivated Localization Microscopy (PALM) Images for Quantitative Measurements

**DOI:** 10.1371/journal.pone.0051725

**Published:** 2012-12-12

**Authors:** Carla Coltharp, Rene P. Kessler, Jie Xiao

**Affiliations:** Department of Biophysics & Biophysical Chemistry, The Johns Hopkins University School of Medicine, Baltimore, Maryland, United States of America; Julius-Maximilians-University Würzburg, Germany

## Abstract

Localization-based superresolution microscopy techniques such as Photoactivated Localization Microscopy (PALM) and Stochastic Optical Reconstruction Microscopy (STORM) have allowed investigations of cellular structures with unprecedented optical resolutions. One major obstacle to interpreting superresolution images, however, is the overcounting of molecule numbers caused by fluorophore photoblinking. Using both experimental and simulated images, we determined the effects of photoblinking on the accurate reconstruction of superresolution images and on quantitative measurements of structural dimension and molecule density made from those images. We found that structural dimension and relative density measurements can be made reliably from images that contain photoblinking-related overcounting, but accurate absolute density measurements, and consequently faithful representations of molecule counts and positions in cellular structures, require the application of a clustering algorithm to group localizations that originate from the same molecule. We analyzed how applying a simple algorithm with different clustering thresholds (*t_Thresh_* and *d_Thresh_*) affects the accuracy of reconstructed images, and developed an easy method to select optimal thresholds. We also identified an empirical criterion to evaluate whether an imaging condition is appropriate for accurate superresolution image reconstruction with the clustering algorithm. Both the threshold selection method and imaging condition criterion are easy to implement within existing PALM clustering algorithms and experimental conditions. The main advantage of our method is that it generates a superresolution image and molecule position list that faithfully represents molecule counts and positions within a cellular structure, rather than only summarizing structural properties into ensemble parameters. This feature makes it particularly useful for cellular structures of heterogeneous densities and irregular geometries, and allows a variety of quantitative measurements tailored to specific needs of different biological systems.

## Introduction

Localization-based superresolution methods such as Photoactivated Localization Microscopy (PALM), Stochastic Optical Reconstruction Microscopy (STORM), and direct STORM (dSTORM) utilize the stochastic switching of fluorophores between dark and bright emission states to visualize fluorophore-labeled single molecules sequentially from the ensemble pool [1]–[3]. The position of each molecule is localized with nanometer precision by fitting its spot intensity profile to a two-dimensional Gaussian function that approximates its point spread function (PSF) [4]. A superresolution image is then generated by overlaying the detected molecule positions. From the superresolution image or the original list of molecule positions, one can quantitatively measure the dimensions and molecule density (number of molecules per unit area) of nanometer-scale cellular structures.

Previously, we and other groups employed superresolution imaging to characterize the cytokinetic “Z-ring” formed by the tubulin homolog FtsZ in bacterial cells [5]–[7]. At the onset of bacterial cell division, a subpopulation of cytoplasmic FtsZ molecules localizes to the midcell membrane, and forms a ring-like structure to initiate the assembly of the division complex [8]. Understanding the structure of the Z-ring will provide important insight into the constriction force generation mechanism of bacterial cell division. Toward this goal, we used PALM data to measure the *in-vivo* dimensions and molecule density of the Z-ring to deduce the arrangement of FtsZ protofilaments inside the ring [7]. Such quantitative structural information is difficult or impossible to obtain using conventional light microscopy, especially in small bacterial cells.

Other PALM and (d)STORM studies have resolved the dimensions of structures such as the ParA bundles that segregate *Caulobacter crescentus* chromosomes [9], microtubule filaments [3], [10], actin filaments [3], clathrin pits [11], budding HIV-1 virions [12], [13], clusters of bacterial histone-like proteins [14], and membrane receptor clusters [15], [16]. Furthermore, molecule density measurements have allowed investigation of the maturation [17] and mechanical load [18] of focal adhesions, the assembly of microclusters following T cell activation [15], [16], [19], assembly of bacterial chemotaxis clusters [20], and the reorganization of membrane protein clusters upon cholesterol addition or depletion [21]. The growing body of examples highlights the great potential of obtaining quantitative information such as structural dimensions, stoichiometry, and molecule density from localization-based superresolution studies. However, care must be taken to ensure the reliability of superresolution data. Many factors influence the resulting superresolution images, including the method of image reconstruction [22], acquisition conditions [23], [24], and movement of the structure of interest. In this work, we focus on one issue that significantly affects the accuracy of quantitative density measurements in PALM imaging – fluorophore blinking.

**Figure 1:**
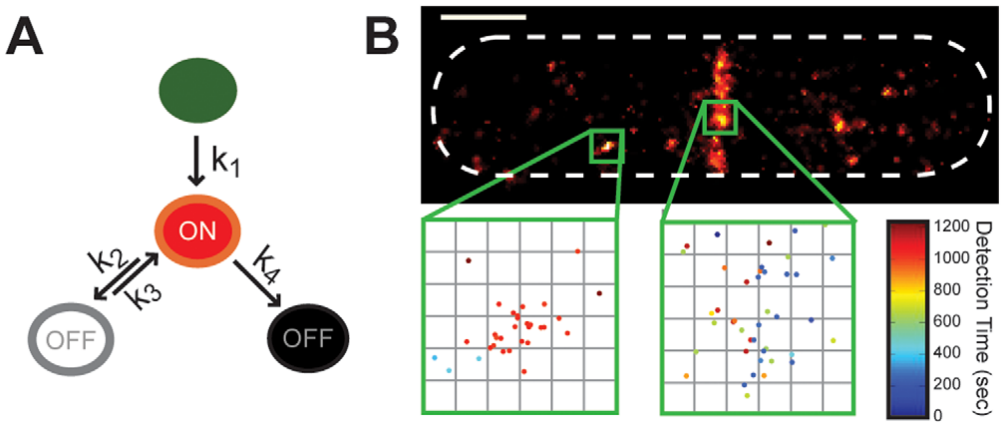
Fluorophore blinking affects superresolution image quality. (A) Simplified kinetic scheme of a photoactivatable fluorophore such as mEos2. The fluorophore is irreversibly photoactivated with rate constant k_1_, can transiently access a nonfluorescent state with rate constant k_2_, return to the fluorescent state with rate constant k_3_, and irreversibly photobleach with rate constant k_4_. (B) Superresolution image of an *E. coli* cell expressing FtsZ-mEos2 generated with conventional clustering thresholds: spots within 167 nm (1 camera pixel) and 50 ms (1 frame) of each other were grouped together and plotted once. The cytoplasmic cluster (left inset) consists of spots detected very closely in time, suggesting that they came from the same fluorophore, whereas a dense section inside the Z-ring (right inset) contains spots detected throughout the experiment. Scale bar, 500 nm. Inset grid size, 30 nm.

Many photoactivatable fluorescent proteins have recently been shown to fluoresce intermittently, or “blink”, once activated [25]–[27]. In Figure 1A we show a simplified reaction scheme of the intermittent fluorescing behavior of a generic photoactivatable fluorescent protein. An activated fluorophore can reversibly transit between a nonfluorescent state (white) and a fluorescent state (red), or can be irreversibly photobleached (black) from the fluorescent state. Intermittent fluorophore blinking leads to overcounting artifacts where single molecules are represented multiple times, resulting in images that are often characterized by a punctate quality [21], [25], [26], [28].

To illustrate this point, we show in Figure 1B a superresolution image of an *Escherichia coli* cell expressing the FtsZ protein fused to mEos2, a photoactivatable fluorescent protein and a popular choice for PALM imaging [29]. In addition to the expected midcell Z-ring formed by FtsZ, the image shows bright cytoplasmic clusters of FtsZ-mEos2. The time-coded insets show that some of these clusters are comprised of spots that were detected very closely in time, in contrast to a region inside the Z-ring where multiple localizations were randomly detected in time. As the activation rate of new fluorophores is usually kept very low in superresolution imaging, these multiple localizations in the cytoplasmic cluster likely come from the same mEos2 molecule rather than multiple molecules.

In various attempts to account for photoblinking so that each molecule in a superresolution image is represented only once, several studies have employed a simple clustering algorithm to group multiple localizations of a single molecule based on the occurrence of these localizations within certain time and distance thresholds (*t_Thresh_* and *d_Thresh_*) – if two localizations occur within *t_Thresh_* and *d_Thresh_*, they likely come from the same molecule and should be grouped together [20], [21], [26], [30], [31]. Each group is then plotted only once in the superresolution image. This type of clustering algorithm was employed in the original demonstrations of STORM and PALM, where fluorescence spots detected within one camera pixel (*d_Thresh_*  = 167 nm for our system) and separated by up to one or three frames, respectively, were grouped together [1], [10]. Generous values for both thresholds can ensure that multiple molecule localizations are grouped together, but may result in false grouping of separate molecules and underestimation of molecule density. It was noted that these thresholds should be optimized based on each fluorophore's photophysical properties [1], [28], but a procedure to identify optimal *t_Thresh_* and *d_Thresh_* values that accurately group localized spots has yet to be developed.

In this study, we analyzed experimental and simulated data to determine how photoblinking kinetics and threshold selection affect the dimension and molecule density measurements described above. We performed these analyses on superresolution images of fixed *E. coli* cells expressing FtsZ-mEos2, whose heterogeneous distribution between midcell and cytoplasmic populations presents a challenging, but ideal, test case for the clustering algorithm. We explained and corroborated the observed effects using datasets in which fluorophore blinking was simulated according to the kinetic scheme shown in Figure 1A.

Our simulations confirmed that blinking-related overcounting increases absolute density measurements, but measurements of dimension and relative density are not affected. We also determined that applying a clustering algorithm can result in inaccurate measurements of both density and dimension measurements unless proper threshold values are chosen and fluorophore activation is slow enough to allow accurate grouping of molecule localizations.

From the above analyses, we identified a simple method for determining the maximum activation rate for a given sample and for selecting optimal *t_Thresh_* and *d_Thresh_* values to generate reliable superresolution images. We further confirmed the generality of this method using simulations of circular clusters of various sizes, which yielded similar results to the FtsZ simulations, suggesting that these principles are applicable to the measurements of cellular structures of different geometries.

In contrast to other recent methods that account for photoblinking [21], [26], which summarize structural properties with global parameters, our optimized clustering method provides the full, corrected, superresolution image and list of molecule positions, which can be quantified in various ways depending on the unique properties of different biological structures. This flexibility is especially useful for heterogeneous cellular structures with regions of high and low molecule density or unusual geometries. Lastly, because our method is a simple improvement of existing PALM clustering algorithms, its implementation is simple and straightforward.

## Results

### Dimension measurements are not affected by fluorophore blinking, but can be affected by applying a clustering algorithm

**Figure 2:**
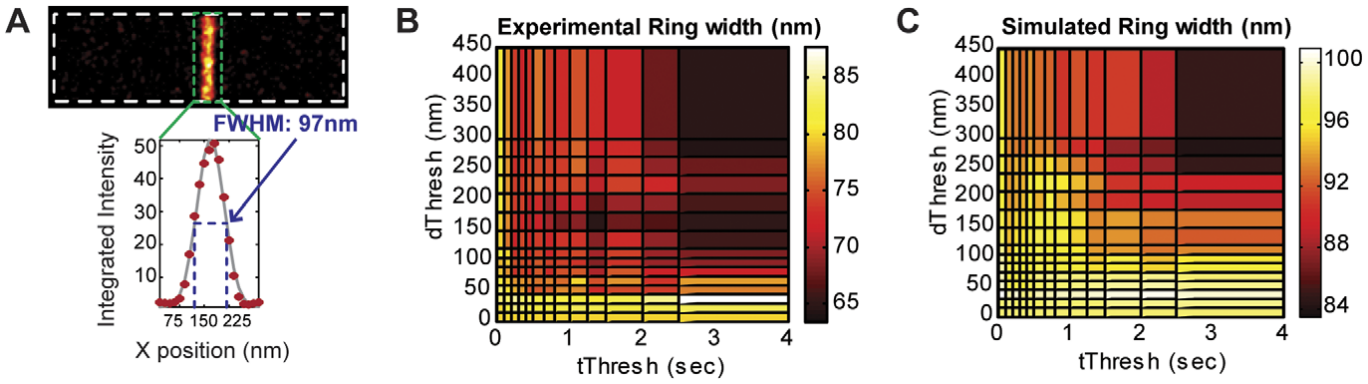
Effects of threshold selection on Z-ring width measurement. (A) Representative Z-ring width measurement of a simulated image where each molecule is only represented once. The intensity along the short axis of the cell is projected onto one dimension (red circles), and then fit to a Gaussian distribution (gray line). The FWHM (97 nm, blue dotted line) is calculated as 2.35*σ, where σ is the fitted Gaussian standard deviation. (B) and (C) Z-ring width values (indicated by the heat map) calculated from images generated by applying different threshold pairs for an experimental dataset (B) and a simulated dataset (C). The simulated dataset was generated using the following parameters: N_total_  = 2000 (50% in the Z-ring), σ = 15 nm, <n_blink_>  = 2, <τ_off_>  = 1 frame, <τ_on_>  = 1 frame, <τ^0^_act_>  = 5 frames (1 frame  = 50 ms). The Z-ring width calculated from the reference image (A), where each molecule is represented only once, is 97 nm, which is similar to the measurements made from images constructed using low values of *t_Thresh_* or *d_Thresh_*.

Structural dimensions are often measured from superresolution images by fitting feature intensity (or density) profiles to Gaussian distributions and extracting the full width at half maximum (FWHM) [7], [10], [14]. Figure 2A shows an example of how the width of the Z-ring is measured in this way. It is important to note that the measured FWHM of a feature is a convolution of the actual feature dimension with the achievable spatial resolution often described as a Gaussian function, the standard deviation of which is determined by the positional variation of multiple localizations of the same molecule (see Text S1 for discussion of spatial resolution). The observed FWHM can be further broadened by the image construction method, which often entails plotting each localized molecule as a Gaussian spot with standard deviation equal to the localization precision [22]. Nevertheless, even with these statistical broadening effects, the FWHM serves as a convenient measurement for structural dimension comparison. Furthermore, with prior knowledge of the distribution of molecules within a structure, the true dimension can be deconvolved [10]. For simplicity, in the following analyses we quantify Z-ring width as the apparent FWHM from a single Gaussian fit.

To determine the effect of threshold selection on dimension measurement, we applied the clustering algorithm (see Methods and Figure S1 for algorithm schematic) with varying *t_Thresh_* and *d_Thresh_* values to both experimental and simulated data. Using a single experimental dataset obtained with FtsZ-mEos2, we generated a series of superresolution images using different threshold pairs and then measured the Z-ring width in each image (Figure 2B). Across the investigated threshold range (*t_Thresh_*: 0–4 sec, *d_Thresh_*: 0–450 nm), Z-ring width varied approximately 1.5-fold (60–90 nm) with larger threshold values resulting in smaller width measurements. We observed the same trend with measurements made from a simulated dataset (Figure 2C) where molecules are allowed to blink according to the kinetic scheme in Figure 1A (see Methods for simulation procedure and parameters). To compare these measurements with the “true” Z-ring width, we used the simulated dataset to generate a reference image in which each molecule is only localized once. The Z-ring width measured from this reference image (97 nm) represents the expected value from an image with no photoblinking artifacts. Widths measured from images generated with most small threshold pairs (*t_Thresh_* <1 s and *d_Thresh_* <100 nm) were within the 95% confidence interval for the fitted Z-ring width in the reference image (94 nm –100 nm), while extremely high threshold values yielded much smaller Z-ring width measurements.

The general decrease in Z-ring width measurement at large thresholds is due to false grouping of localizations that originated from multiple molecules, which affects dimension measurements in two ways. First, because the convolved Z-ring profile is described by a Gaussian distribution, groups that are comprised of multiple molecules have centroid positions weighted toward the center of the Z-ring. Plotting these centroid positions tightens the spatial distribution of the Z-ring and results in smaller width measurements. Second, very large thresholds can cause insufficient sampling of the Z-ring, resulting in further reduced width measurements.

We note that when no clustering is applied (*t_Thresh_* and *d_Thresh_* values of 0), the Z-ring width measured from the simulated data approximates that measured from the reference image. This can be understood if multiple localizations of the same molecule result in an increased amplitude but unchanged standard deviation of the Z-ring Gaussian distribution. This observation is consistent with previous studies where it was shown that multiple localizations of fluorophores improves the statistical sampling of underlying structures and can increase the apparent spatial resolution of a superresolution image [24], [32], [33]. Therefore, reliable dimension measurements can be obtained from a superresolution image that has not been processed using a clustering algorithm, as long as the structure of interest is sampled sufficiently. It is also evident that, if a clustering algorithm is applied, the thresholds should be low enough to avoid underestimation due to false clustering.

### Effects of fluorophore blinking and clustering thresholds on density measurements

Counting the number of fluorescent molecules detected within a given structure provides information about the molecule density of the protein of interest when labeling stoichiometry and the fraction of fluorophores detected are taken into account. In a PALM experiment, the fraction of detected fluorophores is limited because not every fluorescent molecule can be activated and visualized on a finite timescale, and because a substantial fraction (up to 20%) may not be activatable due to misfolding or incomplete fluorophore maturation [34], [35]. However, even with these limitations, molecule counting can still inform structural models by providing a lower bound of molecule density, which can suggest a minimum number of layers or subunits within a structure.

Fluorophore blinking results in erroneous amplification of fluorophore density due to repeat localizations. Below we explore how *t_Thresh_* and *d_Thresh_* selection affects three types of measurements: mean density, relative density, and density distribution measurements. Mean density is a bulk measurement of the average number of molecules per unit area; relative density reports the fraction of molecules confined to a particular region of the structure; and the density distribution is described by the histogram of number of molecules detected per unit area. Fluorophore blinking amplifies both mean density and density distributions, which are absolute measurements of molecule counts, but relative density, which is the ratio between molecule counts in different regions, should not be affected if all molecules have the same ensemble blinking properties.

#### Mean density measurements are affected by fluorophore blinking and by applying a clustering algorithm

**Figure 3:**
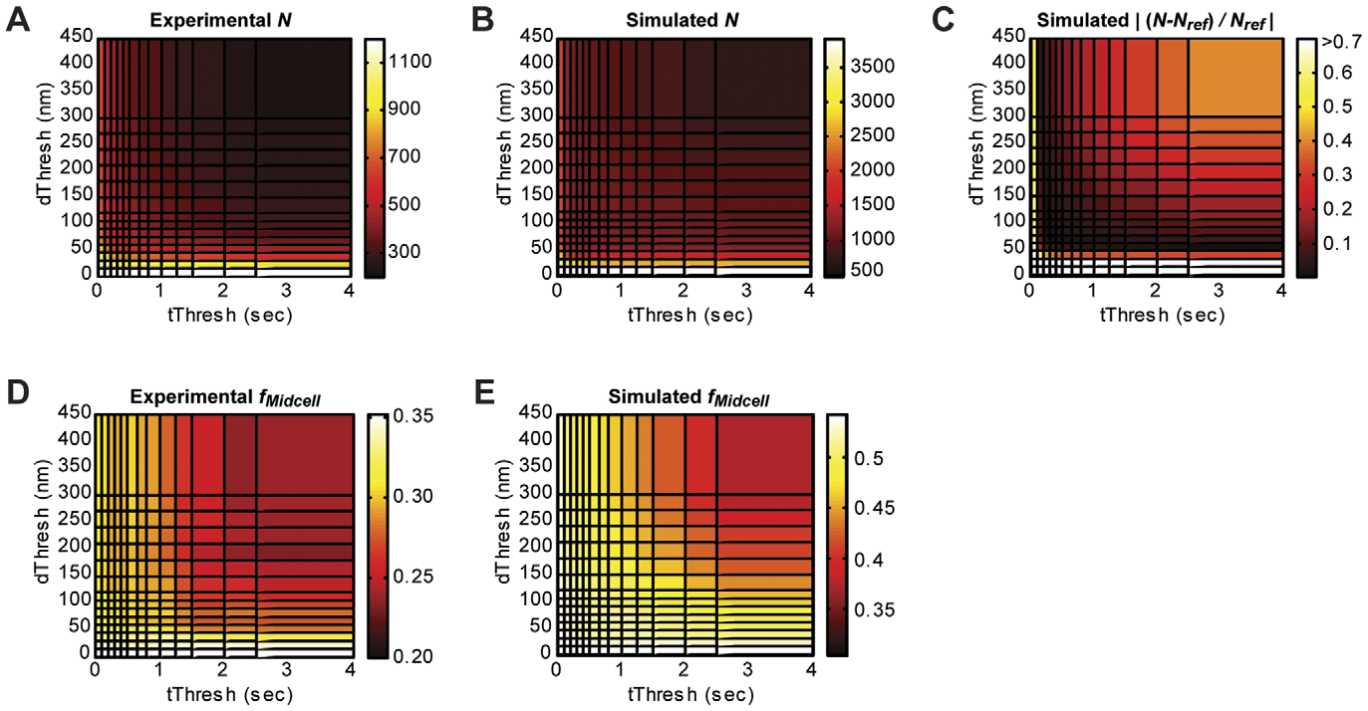
Effects of threshold selection on mean and relative molecule density. (A) and (B) Total number of molecules, *N*, in images generated by applying different threshold pairs to an experimental dataset (A) and a simulated dataset (B). (C) Fractional difference |(*N-N_ref_)/N_ref_*| between each reconstructed simulated image and the number of molecules in the reference simulated image (*N_ref_*  = 1248). Images with small fractional differences (dark areas) are generated from threshold pairs found along two intersecting valleys. (D) and (E) Fraction of molecules located at the midplane (*f_midcell_*) in images generated by applying different threshold pairs for an experimental dataset (D) and a simulated dataset (E). In the reference image, *f_midcell_*  = 0.53, which is most similar to the values calculated from images generated using low values of both *d_Thresh_* and *t_Thresh_*. Datasets analyzed are the same as those shown in Figure 2.

Using the same experimental and simulated datasets shown in the dimension measurement analysis in Figure 2, we examined the effect of t_Thresh_ and d_Thresh_ selection on mean density (molecules per unit area) by analyzing how the total number of molecules (N) in the same cell area varies with different t_Thresh_ and d_Thresh_ pairs. Both experimental (Figure 3A) and simulated (Figure 3B) datasets display the same trend: larger thresholds yield lower mean densities (N/cell area) because more spots are grouped together. For the simulated dataset, we determined the fractional difference of N from the true value obtained from the reference image, |(N-N_ref_)/N_ref_|, which is plotted in Figure 3C. We found that reasonably accurate mean density measurements (<10% difference from N_ref_) can be achieved by multiple threshold pairs along two valleys that intersect at t_Thresh_ ≈ 0.4 s and d_Thresh_ ≈ 60 nm.

#### Relative density measurements are not affected by fluorophore blinking, but can be affected by applying a clustering algorithm

Next, we examined the effect of t_Thresh_ and d_Thresh_ selection on relative density measurements by measuring f_midcell_, the fraction of molecules detected in the Z-ring relative to the whole cell. Increasing values of both t_Thresh_ and d_Thresh_ resulted in smaller f_midcell_ values for both experimental (Figure 3D) and simulated (Figure 3E) datasets. Comparison with the f_midcell_ value calculated from the reference image (0.53) revealed that images generated with smaller thresholds yielded reasonably accurate relative density measurements (<10% difference for t_Thresh_ <1 s and d_Thresh_ <105 nm). This is because molecules within and outside the structure of interest have the same blinking properties, allowing the effect of multiple molecule localizations to cancel out in a relative measurement. The underestimation of f_midcell_ for larger threshold values is due to the greater likelihood of falsely grouping molecules in denser regions such as the Z-ring. These results confirm that, as with the Z-ring width measurement, relative density can be accurately measured from a superresolution image that has not been processed using a clustering algorithm (t_Thresh_ and d_Thresh_ equal to 0), as long as the structure of interest is sampled sufficiently.

#### Molecule density distributions are affected by fluorophore blinking and by applying a clustering algorithm

**Figure 4:**
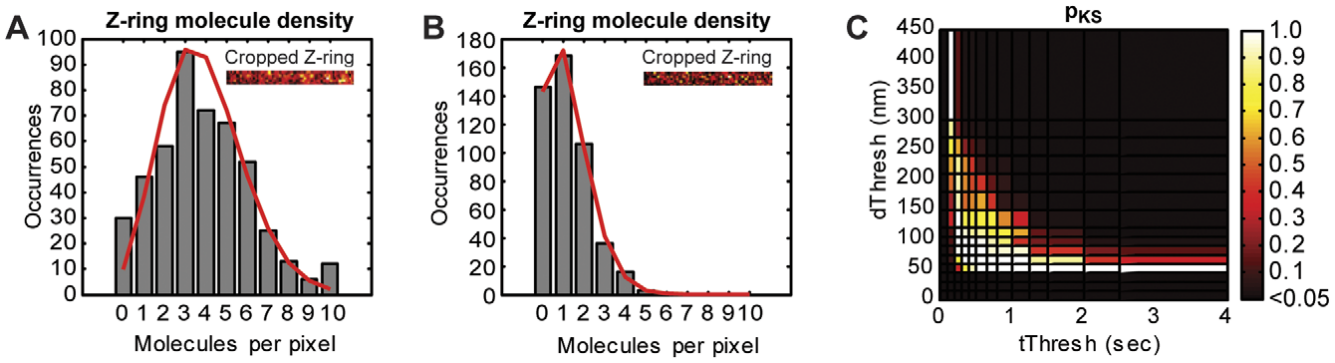
Effects of threshold selection on molecule density distribution in the Z-ring. (A) Histogram (gray bars) of molecules per pixel (15 nm ×15 nm) inside the Z-ring of a simulated image that was not processed with a clustering algorithm. (B) Histogram (gray bars) of molecules per pixel of the corresponding reference image, where each molecule is represented only once. Poisson distributions simulated with the sample means, 3.9 (A) and 1.2 (B) molecules per pixel, are shown in red. The ratio of mean values reflects the localization of each molecule approximately three times due to the simulated photoblinking kinetics (<n_blink_>  = 2, <τ_off_>  = 1 frame, <τ_on_>  = 1 frame). Poisson goodness-of-fit tests resulted in p_GOF_  = 0 for distribution in (A), suggesting that blinking results in deviations from a Poisson density distribution (p_GOF_  = 0.74 for the reference distribution in (B)). Insets show the cropped Z-ring regions used to generate the histograms. (C) p-values from the KS-test when the molecule density distribution of the Z-ring generated by the reference image (B) is compared with distributions in images generated with different threshold pairs. Distributions that resulted in p_KS_ >0.05 are not significantly different from the distribution in the reference image. Dataset analyzed is the same simulated dataset shown in Figure 2–3.

While mean and relative molecule densities describe global structural properties, the molecule density distribution provides additional information about the uniformity or heterogeneity of molecule positions within a structure. If molecules are uniformly distributed throughout the structure, the molecule density histogram should be well described by a Poisson distribution. Figure 4A shows the histogram of molecules detected per superresolution pixel (15 nm ×15 nm) within the Z-ring of the simulated dataset before any clustering algorithm is applied. A Pearson Χ^2^ goodness-of-fit test [36] shows that this distribution deviates from a Poisson distribution significantly (p_GOF_  = 0). This seemingly heterogeneous distribution of molecules inside the Z-ring, as judged by the significant deviation from Poisson distribution, is actually caused by multiple localizations of single molecules due to photoblinking. Figure 4B shows that the Z-ring molecule density of the corresponding reference image where each molecule is only localized once can be described by a Poisson distribution adequately (p_GOF_  = 0.74).

To identify the clustering thresholds that generate the correct molecule density distributions, we compared the Z-ring molecule density distributions generated with different *t_Thresh_* and *d_Thresh_* pairs to the distribution from the reference image using the Kolmogorov–Smirnov (KS) test [37]. As with mean density, density distributions are similar to the reference distribution (*p_KS_* >0.05) along two intersecting ridges (Figure 4C), indicating that the thresholds in this region generate images that most faithfully represent the true molecule distributions.

### Multiple threshold pairs can result in accurate measurements of both dimension and density measurements

**Figure 5:**
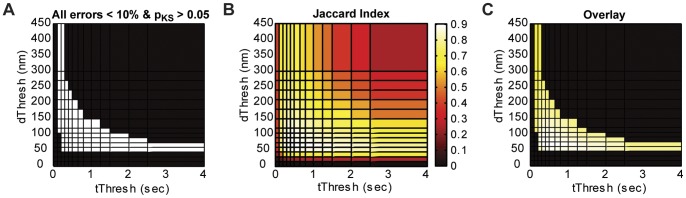
Accuracy of images generated with different threshold pairs. (A) Region of threshold space (white squares) that resulted in <10% difference from the reference measurements of Z-ring width, *N*, and *f_midcell_*, and that yielded Z-ring density distributions not significantly different from the reference distribution (pKS >0.05). (B) Jaccard index values at each threshold pair. Higher Jaccard index values indicate more accurate single-molecule clustering. (C) The peak of the Jaccard index plot (B, white squares) is within the region where all four quantitative measurements are within 10% of the reference measurements (A). Dataset analyzed is the same simulated dataset shown in Figure 2–4.

We have shown that the mean molecule density and density histogram measurements are significantly affected by fluorophore blinking, while dimension and relative density measurements are not. However, all four measurements are affected by the selection of *t_Thresh_* and *d_Thresh_*. We have also shown that each type of measurement can be made within a reasonable range of the true value if correct threshold values are chosen. To find the threshold region that simultaneously represents all four measurements with reasonable accuracy, we combined the relative errors obtained at different threshold pairs for each measurement made from the simulated images described in Figures 2, 3, 4. Figure 5A shows the region (white squares) where Z-ring width, *f_midcell_*, and *N* measurements are within 10% of the reference values and density distributions were not significantly different from the reference distribution (*p_KS_* >0.05).

### The Jaccard Index identifies images that are accurate on both the ensemble and single-molecule level

The combined error analysis selected a limited threshold range rather than a unique, optimal threshold pair. While this range of thresholds can satisfy all investigated quantitative measurements simultaneously, some threshold pairs in this range may not generate accurate superresolution images in which the molecule counts and positions are correctly identified. This is because each of the measurements described above quantifies an ensemble structural property and may not be sensitive to errors at the individual molecule level.

To find the optimal threshold pair that not only provides reliable quantitative measurements, but also an accurate image at the single-molecule level, we further assessed the clustering accuracy of each threshold pair using the Jaccard index [38], [39]. The Jaccard index compares how frequently localizations of the same molecule are correctly grouped together (true positives), how frequently localizations of different molecules are incorrectly grouped together (false positives), and how frequently localizations from the same molecule are not grouped together (false negatives) (see Methods for details). The Jaccard index, therefore, reflects clustering accuracy at the individual molecule level; higher Jaccard index values indicate more accurate grouping, and consequently more accurate image reconstruction.

We calculated the Jaccard Index as a function of *t_Thresh_* and *d_Thresh_* (Figure 5B) for the simulated dataset described above and observed a clear peak at *t_Thresh_*  = 0.4 s and *d_Thresh_*  = 60 nm. This peak position is within the optimal threshold region selected by the combined measurement error plot (Figure 5A). This agreement, illustrated by the overlay plot in Figure 5C, confirms that the *t_Thresh_* and *d_Thresh_* pair that most correctly groups localizations (maximizes Jaccard index) also generates an image that provides accurate quantification of dimension and density.

### Structural geometry does not affect the positions of the optimum threshold pair and Jaccard index peak

**Figure 6:**
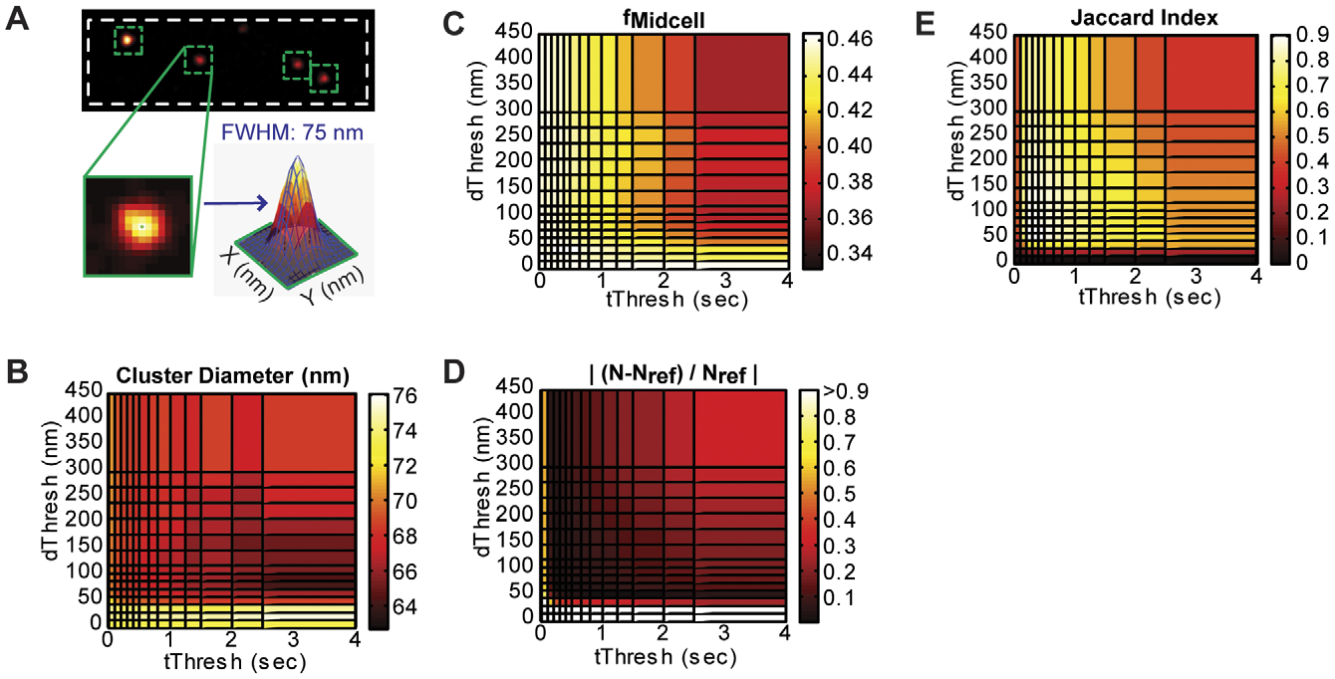
Quantitative measurements of a simulated cluster dataset. (A) Representative cluster diameter measurement for a reference image with no repeat localizations. Each cluster is identified by eye, and then fit to a two-dimensional, symmetrical Gaussian distribution (blue mesh). The cluster diameter is measured as the FWHM, calculated as 2.35*σ, where σ is the fitted Gaussian standard deviation. The average FWHM of these four clusters is 74±1 nm. (B) Cluster diameter values (average of four clusters) calculated from images generated by applying different threshold pairs to the same simulated dataset. The measured diameters decrease with increasing threshold values, similarly to the Z-ring width measurement. (C) The fraction of molecules located in clusters (*f_cluster_*) is most similar to that measured in the reference image (0.47) for low values of both *d_Thresh_* and *t_Thresh_*. (D) As with the Z-ring simulation, fractional difference between each reconstructed image and the number of molecules in the reference image (*N_ref_*  = 1212) is lowest along two intersecting valleys. (E) The Jaccard index peak position for the cluster simulation is similar to that in the Z-ring simulation where identical kinetic parameters were used (Figure 5B). This simulated dataset was generated using the following parameters: N_total_  = 2000 (50% in clusters), <molecules/cluster>  = 200, FWHM_cluster_  = 50 nm, σ  = 15 nm, <n_blink_>  = 2, <τ_off_>  = 1 frame, <τ_on_>  = 1 frame, <τ^0^_act_>  = 5 frames (1 frame  = 50 ms).

To examine whether differences in structural geometry or dimension can alter the observed effects of photoblinking or threshold selection on quantitative measurements, we simulated another type of cellular structure: circular clusters of various sizes (see Methods for simulation details). Figure 6 shows the quantitative analyses of one simulation that was generated with the same photoblinking parameters as the FtsZ simulation discussed in Figures 2, 3, 4, 5, but with molecules distributed among several clusters (Figure 6A) rather than a single Z-ring.

Despite the structural difference, the cluster simulation showed a similar trend when different threshold pairs are applied. Figure 6B and Figure 6C reproduce the general trends that larger threshold values result in smaller dimension measurements and relative density measurements, respectively. The intersection of valleys in the|(*N-N_ref_*)*/N_ref_*| plot (Figure 6D) and the Jaccard index peak (Figure 6E) coincide with those observed for the Z-ring simulation (Figure 3C and 5B, respectively). The shared peak positions between simulations with different underlying structure suggests that structural geometry does not affect the position of the optimum thresholds (see Discussion).

### Low measurement error is not sufficient to guarantee high Jaccard index

**Figure 7:**
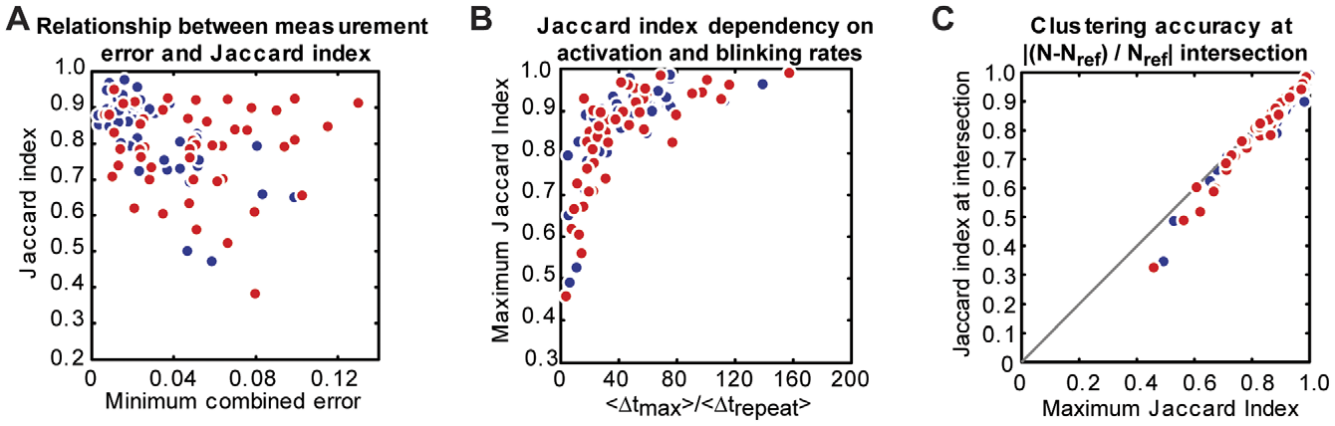
Relationship between Jaccard index, measurement error, and activation rate across different simulated datasets. (A) Minimum combined measurement error, ε_all_, for each dataset plotted against and the corresponding Jaccard index value. ε_all_ was defined as the worst fractional measurement error of the three bulk measurements: *N, f_midcell_*, and ring width when compared to the reference image. Images with low measurement error do not always correlate with high clustering accuracy (Jaccard index), and thus cannot ensure reliable lists of molecule counts and positions. (B) Maximum Jaccard index plotted against the ratio of the average time between localizations in the 255 nm ×255 nm maximum density region, *Δt_max_*, and the average time between repeat localizations of the same molecules, *Δt_repeat_*, calculated for each simulated dataset. Simulations with higher ratios of *Δt_max_*/*Δt_repeat_* result in higher Jaccard index values. (C) Comparison of maximum Jaccard index with Jaccard index identified at the intersection of the |(*N-N_ref_*)*/N_ref_*| plot for each simulated dataset. The two values agree well when the maximum Jaccard index is greater than 0.8. Simulation parameters can be found in Table S1 and S2. In all plots, Z-ring simulations are shown in blue and cluster simulations are shown in red.

To further illustrate the relationship between Jaccard index and measurement error under a variety of conditions, we generated simulated datasets with different structural geometries, molecule densities, fluorophore blinking properties, and activation rates (see Table S1 and S2 for parameter ranges). For each simulation, we calculated the combined measurement error, *ε_all_* (worst fractional error among the three bulk measurements: *N, f_midcell_* or *f_cluster_*, and ring width or cluster diameter), resulting from each threshold pair. Then, we identified the minimum-error threshold pair that yielded the lowest *ε_all_*. In Figure 7A, we plot the relationship between *ε_all_* generated by the minimum-error threshold pair and the corresponding Jaccard Index achieved by that threshold pair. Across a large range of fluorophore blinking properties and activation rates examined, most Z-ring (blue) and cluster (red) simulations could generate images with low measurement error (<10%) using at least one threshold pair. However, not all of these images achieved high accuracy at the single molecule level (Jaccard index >0.8). This result suggests that achieving accurate ensemble measurements is not sufficient to ensure that the corresponding list of molecule positions is reliable.

### Fluorophore blinking kinetics and activation rate determine achievable accuracy in superresolution image reconstruction

As shown by the region where the maximum Jaccard index is <0.8 in Figure 7A, some simulations could not yield high Jaccard indices regardless of threshold selection. This observation indicates that some combinations of fluorophore blinking properties and activation rates do not allow accurate image reconstruction using the clustering algorithm. We reason that when the probability of molecule activation within a diffraction-limited area (255×255 nm^2^ for mEos2 in our imaging condition) becomes comparable to the probability that a molecule blinks back on, it is difficult for the clustering algorithm to discriminate a repeat localization of an activated molecule from the first localization of a new molecule, resulting in separate molecules falsely grouped as single molecules more frequently.

To understand what combination of fluorophore blinking rate and activation rate ensures a high achievable accuracy in superresolution image reconstruction, we employ two experimentally-measureable parameters, *Δt_max_* and *Δt_repeat_*. We define *Δt_max_* as the average time between subsequent localizations of all molecules within the diffraction-limited region of highest molecule density in a superresolution image. *Δt_max_* can be easily calculated by dividing the total imaging acquisition time by the number of localizations detected in the maximum density region (see Methods). *Δt_max_* therefore increases when activation is slower given the same fluorophore properties, and serves as a convenient measurement of activation rate. We define *Δt_repeat_* as the average time between subsequent localizations of the same molecule, which is determined by fluorophore blinking kinetics, and can be measured from *in vitro* or *in vivo* experiments where single fluorophores are clearly identifiable (Figure S2A).

To explore the relationship between these two parameters and achievable Jaccard index, we calculated *Δt_max_* and *Δt_repeat_* for the simulations analyzed in Figure 7A and plotted the maximum Jaccard index achieved for each simulation against the ratio of *Δt_max_*/*Δt_repeat_* (Figure 7B). Simulations of both FtsZ (blue) and clusters (red) display a clear increase in maximum Jaccard index with increasing *Δt_max_*/*Δt_repeat_*, such that accurate clustering (Jaccard index >0.8) can be achieved when the ratio is kept above 40, regardless of the underlying structure. This observation provides a simple criterion for screening imaging conditions, specifically activation rates, in order to reconstruct a highly accurate superresolution image.

### Identifying the optimal threshold pair from an experimental dataset

We have shown that one can easily identify the optimal *t_Thresh_* and *d_Thresh_* pair from the plot of Jaccard indices at various *t_Thresh_* and *d_Thresh_* values (Figure 5B and 6E). However, the Jaccard Index cannot be calculated for experimental data where the photoblinking behavior of individual molecules is unknown. The overlap of the intersection of valleys in the |(*N-N_ref_*)*/N_ref_*| plot (Figure 3C and 6D) with the Jaccard peak position (Figure 5B and 6E), however, may allow the identification of the optimal thresholds using an experimental observable.

To examine the correlation between the intersection of the valleys in the |(*N-N_ref_*)*/N_ref_*| plot and the threshold pair that gives rise to the peak Jaccard index value, we compared the maximum Jaccard index of different simulations with the Jaccard index achieved using the threshold pair at the intersection of valleys in the |(*N-N_ref_*)*/N_ref_*| plot identified by eye (see Figure S3 for examples of intersection identification). Figure 7C shows that the maximal Jaccard index and the Jaccard index achieved at the intersection are in excellent agreement when the maximal Jaccard index is greater than 0.8. At these high values, the Jaccard index peaks are in general broader, such that threshold pairs nearby the peak provide similar clustering accuracy. At low maximum Jaccard indices, the peak becomes sharper, leading to higher variability in achieving the maximum value by visual inspection. These results indicate that near-optimal threshold values can be identified from the intersection of the valleys in the |(*N-N_ref_*)*/N_ref_*| plot under the ideal experimental conditions described above (*Δt_max_*/*Δt_repeat_* >40, Jaccard index >0.8).

Generation of the |(*N-N_ref_*)*/N_ref_*| plot requires knowledge of the true number of molecules, *N_ref_*. This number can be calculated by dividing the total number of localized spots in the unprocessed image (*N_unprocessed_*) by the average number of localizations per molecule (*α*) under a given imaging condition (illumination power, exposure time) such that *N_ref_*  =  *N_unprocessed_*/*α* [12], [21], [31]. An alternative method developed by Annibale *et al*. extracts *N_ref_* from fitting a semi-empirical equation to the dependence of *N* on *t_Thresh_* at a fixed *d_Thresh_* [26].

**Figure 8:**
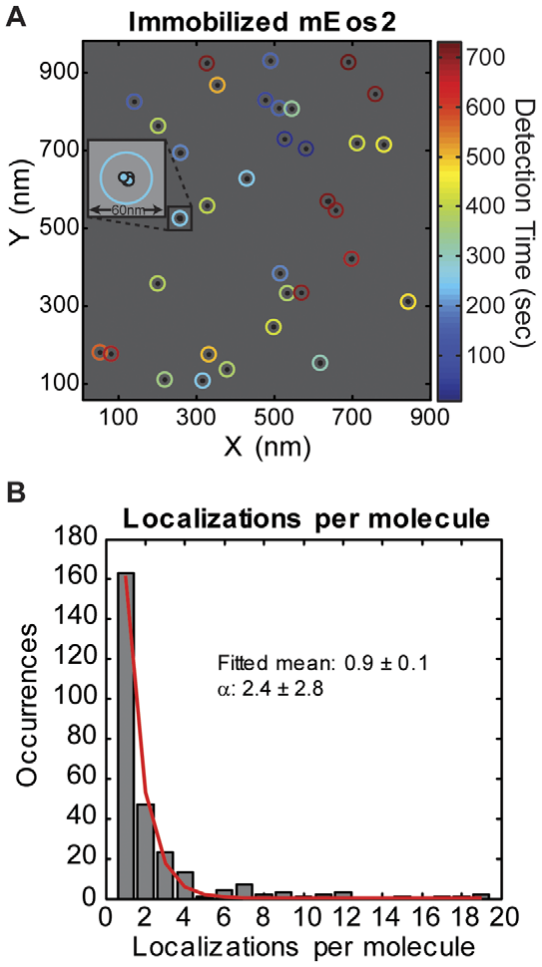
*In vitro* characterization of mEos2. (A) A typical *in vitro* image of purified mEos2 molecules sparsely distributed on a cover glass, acquired using the same PALM imaging condition as the *in vivo* cell sample. All localized positions are indicated by small, filled circles that are colored by detection time. Localizations belonging to the same molecule are enclosed in a larger, open circle, which is colored by the mean detection time of all the enclosed localizations. The inset shows details of a single cluster, which contains four localizations (filled circles with black outlines). (B) Histogram of localizations per molecule from 515 molecules fitted with an exponential distribution (red line), which yielded a mean of 0.9±0.1 localizations per molecule. The value of α (2.4±2.8) represents the mean of observed molecules that lasted at least one frame, and is consequently larger than the fitted mean.

The parameter *α* is a bulk fluorophore property that can be obtained experimentally. We measured *α_mEos2_* by imaging sparsely distributed, immobilized mEos2 molecules *in vitro* under the same buffer and imaging conditions used for fixed cells. Individual mEos2 molecules could be clearly distinguished as well-separated clusters of localizations (Figure 8A). Figure 8B shows that the distribution of localizations per molecule approximates an exponential distribution with a fitted mean of 0.9±0.1. This fitted mean includes the population of molecules that did not last long enough to be detected in one frame, while *α* reflects the number of localizations per molecule that lasted long enough to be detected in at least one frame. Consequently, the value of *α* calculated by dividing the total number of localizations by the number of molecules is larger than the fitted mean. Under our imaging condition, *α_mEos2_* = 2.4±2.8 (s.d., N = 515) for purified mEos2. Furthermore, using fixed *E. coli* cells expressing low levels of untagged mEos2 protein at extremely low activation level, we obtained a similar value (*α_mEos2_* = 2.1±4.3, N = 1228) (Figure S4), suggesting that the blinking properties of mEos2 are similar *in vitro* and in fixed cells under the same buffer conditions. Therefore, we combined the datasets to obtain *α_mEos2_* = 2.2 (Figure S4B), which is within the range of values reported in previous studies under different conditions (*α_mEos2_* = 1–3) [12], [31].

### Application of the optimal threshold pair to experimental data

**Figure 9:**
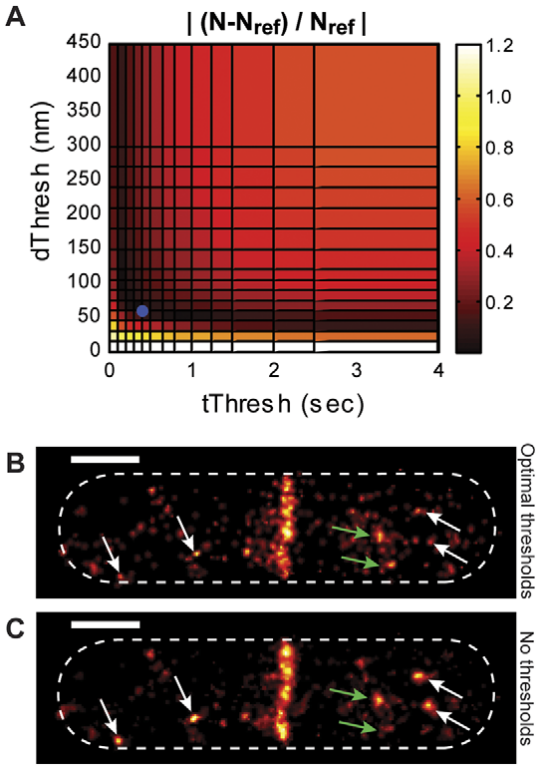
Determination and application of optimal threshold pair to an experimental image. (A) |(*N-N_ref_*)*/N_ref_*| plot of the experimental dataset used in Figure 1–3. The plot was generated using the *N_ref_* number (547) calculated from the measured *α* value from combined *in vitro* and *in vivo* characterizations of mEos2 (Figure 8 and Figures S4). The optimal threshold pair was identified at 0.4 s and 60 nm (blue circle). (B) and (C) Images constructed using the optimal threshold pair (B) and without any clustering algorithm (C). Clusters that are reduced by the optimized clustering algorithm are indicated by white arrows. Clusters that remain bright in the optimal image, which may represent oligomeric states of FtsZ-mEos2, are indicated by green arrows. Scale bars, 500 nm.

Using the experimentally-determined value of *α_mEos2_*, we calculated the true *N_ref_* for the experimental dataset described in Figure 2, 3, which was collected under the same imaging condition as the sparse mEos2 samples (*N_ref_*  =  *N_unprocessed_*/*α_mEos2_* = 1204/2.2 = 547). We then varied both *t_Thresh_* and *d_Thresh_* to generate the plot of |(*N-N_ref_*)*/N_ref_*| (Figure 9A), which is qualitatively indistinguishable from that generated from simulated data (Figure 3C). From this plot we identified the intersection of the two valleys (*t_Thresh_*  = 0.4 s and *d_Thresh_*  = 60 nm) as the optimal threshold pair, which we used to generate the optimal superresolution image (Figure 9B). The images generated using unoptimized clustering thresholds (*t_Thresh_*  = 0.05 s (1 frame) and *d_Thresh_*  = 167 nm) (Figure 1B) and without applying any clustering algorithm (Figure 9C) exhibit several bright cytoplasmic clusters, some of which are almost completely removed in the optimal image (white arrows) while a few remain (green arrows). It was previously suspected that FtsZ forms polymeric structures outside the Z-ring [8], but because of the blinking-related artifacts described above, it has been difficult to interpret the cytoplasmic clusters observed in superresolution images. The optimal image clearly shows selective removal of blinking-related clusters, increasing our confidence in assigning the remaining cytoplasmic clusters to oligomeric states of FtsZ-mEos2 molecules.

In addition to identifying real protein clusters in the optimal superresolution image, we performed the four quantitative measurements described above using the optimal superresolution image. This dataset was acquired over 20 minutes, resulting in *Δt_max_*  = 87 frames. Using the mEos2 characterization data described above, we measured *Δt_repeat_*  = 2.1±3.3 frames (s.d., N = 2072) (Figure S2A), resulting in the ratio *Δt_max_*/*Δt_repeat_*  = 41, which is similar to the criterion provided in Figure 7B (*Δt_max_*/*Δt_repeat_* >40). Consequently, the Z-ring width, *f_midcell_*, and mean molecule density are within 15% of the expected values (Table 1). We also analyzed the Z-ring density distribution from both images using the Poisson goodness-of-fit test. In this case, both the original and the optimal image were well-described by a Poisson distribution (*p_GOF_*  = 0.14 and 0.56, respectively). This result suggests that the blinking behavior of mEos2 does not affect the molecule density distribution of the Z-ring significantly at our level of sampling (<molecules/pixel>  = 0.5 in the Z-ring).

**Table:** **Table1.** Quantitative measurements made from the optimized experimental image.

	Original image	True value	Optimal image	% difference from true value
Ring width	84nm	84nm*	77nm	8%
*f_midcell_*	35%	35%*	30%	14%
*N*	1204	547**	517	5%

* True values for ring width and *f_midcell_* are those measured from the original image. **True value for *N* is the number of molecules in the original image divided by α_mEos2_ (1204/2.2 = 547).

## Discussion

### Selection of localization-based superresolution imaging method

We have shown that reliable dimension and relative density measurements can be made from superresolution images with multiple fluorophore localizations due to photoblinking. For (d)STORM imaging, it has been shown that multiple localizations of single fluorophores can actually enhance image quality because repetitive sampling of the same molecule averages out the stochasticity in individual localizations, leading to convergence on the true molecule position [24], [32], [33]. Therefore, if the goal of a superresolution imaging experiment is to visualize overall structural dimensions and obtain relative density measurements, no clustering algorithm should be applied. In this regard, organic dyes that can undergo hundreds of switching cycles, such as those used in (d)STORM, are especially advantageous. The high photon yields of these dyes also improves the accuracy in localizing single molecules, hence enhancing the spatial resolution of the superresolution image (10–20 nm [10]).

If the goal of a superresolution experiment is to count the number of molecules accurately in a cellular structure so that stoichiometric information of molecular components can be obtained, photoactivatable fluorophores that are irreversibly activated and exhibit only a few localizations before permanent photobleaching are desirable. Photoactivatable fluorescent proteins such as mEos2 yield only a few localizations per molecule and provide the benefit of stoichiometric labeling via genetic fusion to a protein of interest, improving the accuracy of counting molecules. We show that photoactivatable fluorescent proteins can yield accurate density measurements if activation is kept adequately slow and a clustering algorithm with carefully-selected thresholds is applied. Because conditions and fluorophores for (d)STORM are often optimized to yield long dark times between reversible switching events (large values of *Δt_repeat_*) [24], [33], these experiments require prolonged imaging time if the goal is to achieve accurate clustering (see below). Therefore, PALM experiments employing photoactivatable fluorescent proteins such as mEos2, mEos3, and PAmCherry are better suited for molecule-counting than reversibly switchable dyes.

In a live-cell superresolution experiment, an additional consideration for accurate molecule counting is the movement of single molecules. These movements often occur on the millisecond imaging timescale, resulting in single molecules localized at multiple positions throughout a structure. In a similar manner to reversibly switching dyes, this can be beneficial because it allows for faster sampling of the entire structure with fewer fluorophores [40]. However, molecule movement makes application of the clustering algorithm described above difficult because the optimal distance threshold will depend on the molecule's diffusion rate. Therefore, to obtain accurate molecule counting and absolute density within a structure, fixed-cell PALM experiments with carefully optimized fixation protocols that minimize structural aberrations are most suitable. Below we discuss further considerations and limitations in applying the clustering algorithm for fixed-cell PALM experiments.

### Achievable clustering accuracy is dependent on imaging acquisition condition

We have described a method for selecting the optimal values of *t_Thresh_* and *d_Thresh_* based on visual inspection of the |(*N-N_ref_*)*/N_ref_*| plot. The optimal values of *t_Thresh_* and *d_Thresh_* ensure that most repeat localizations are grouped together, minimizing false identification of repeat localizations as separate molecules. However, we have also shown that successful application of the optimized clustering algorithm requires low activation rates, where the probability that a new molecule will be photoactivated nearby an activated molecule before it is photobleached is low. High activation rates result in false grouping of separate molecules.

To minimize false molecule grouping, we have provided the criterion that *Δt_max_* needs to be more than 40-fold longer than *Δt_repeat_* (Figure 7B). For the mEos2 protein characterized in this study, we observed that *Δt_repeat_*  = 2.1 frames in our experimental setup (Figure S2). Consequently, a minimum *Δt_max_* of 84 frames between localizations in the 225±225 nm^2^ square of highest density is required. This criterion can be converted into a maximum detection rate of 0.24 molecules/µm^2^ per frame in the maximum density region (1/0.05 um^2^/84 frames), which also satisfies the conditions previously reported to avoid detection of overlapping fluorescent molecules (<0.5 molecules/µm^2^ per frame on average [23]).

### Physical meaning of optimal threshold values

Under an imaging condition that allows accurate superresolution reconstruction, the optimal values for *t_Thresh_* and *d_Thresh_* are related to the mean off time of fluorophore blinking (<*τ_off_*>) and the spatial resolution, respectively. Longer <*τ_off_*> values require larger values of *t_Thresh_* to accommodate the longer dark times between spots so that most repeat localizations of the same molecule are grouped into the same cluster (Figure S5). Likewise, the *d_Thresh_* value increases with the value of spatial resolution (Figure S6), which is defined as the FWHM of Gaussian-distributed fluorophore localizations around the central molecule position, and determines the average distance between repeat molecule localizations (Text S1). The optimal *d_Thresh_* value is approximately twice the spatial resolution, which is large enough to account for most repeat localizations of the same molecule (Figure S7E). The values obtained for the experimental image described above (*t_Thresh_*  = 0.4 s, *d_Thresh_*  = 60 nm) agree with the <*τ_off_*> timescale of mEos2 blinking kinetics observed in our study (0.10 s, Figure S7B) and previous studies (0.1–0.4 s) [26], as well as the typical spatial resolution of mEos2 molecules in our setup (42 m; Figure S7D). Although this general correlation (Figure S5–6) could also be used to estimate optimal threshold values, substantial variations in corresponding optimal threshold values exist due to other fluorophore photoproperties or structural parameters. Generation of the |(*N-N_ref_*)*/N_ref_*| takes all of these factors into account, while requiring similar effort in fluorophore characterization.

### Fluorophore characterization

Two fluorophore properties need to be determined to implement the method we have described. First, the average time between repeat molecule localizations, *Δt_repeat_*, should be compared with the activation rate to identify imaging conditions that are suitable for accurate superresolution image reconstruction as described above. Second, the average number of observations per molecule, *α*, is required to generate the |(*N-N_ref_*)*/N_ref_*| plot. Both of these parameters should be determined using the same exposure time and excitation intensity as those used for the superresolution imaging. This can be done with either purified fluorophore or, ideally, with a fixed biological sample of low labeling density that is activated extremely slowly. It is also important to characterize a fluorophore under the same buffer conditions used for superresolution imaging because blinking kinetics are highly dependent on a fluorophore's chemical environment [27]. The conditions inside fixed cells should be equilibrated with external buffer conditions, but this is not the case for live cells. Since movement can complicate fluorophore characterization in live cells, we again recommend fixed cells for the purposes of accurate molecule counting and density measurements.

The kinetic scheme that we used to described the blinking behavior of mEos2 is relatively simple (see Methods) and has been previously shown to sufficiently describe mEos2 [26]. Although more complex schemes, in which multiple pathways lead to the observed dark or fluorescent states [28], [41]–[44], may also be applicable to mEos2 and other photoactivable fluorophores, we have verified experimentally that the distributions of *τ_on_*, *τ_off_*, and *n_blink_* observed from individual mEos2 molecules can be approximated well by the single exponential distributions used in our simulation (Figure S7A, B, C).

### Comparison with other superresolution processing algorithms

A few recent methods have addressed some of the effects of photoblinking on superresolution images. The method developed by Annibale *et al*. identifies the correct number of molecules (*N_ref_* ) in a superresolution image by fitting the dependence of *N* on *t_Thresh_* to a semi-empirical function [26]. In addition to providing an unambiguous way to determine the true *N_ref_* in a heterogeneous image, our method provides optimal *t_Thresh_* and *d_Thresh_* values to generate a reliable image that can be quantified in many different ways, and identified a quantitative criterion (*Δt_max_*/*Δt_repeat_* >40) under which optimal image reconstruction is possible.

Other studies have applied pair correlation analyses to images of membrane clusters to extract mean density and cluster size [21], [45]. By analyzing correlation functions of distances between detected spots, these studies have elegantly determined the relative contribution of blinking and true molecular interactions to the observed clusters in their superresolution images. Because the pair correlation analysis does not require classification of each localized spot, it can accommodate faster activation rates than the method we have described. However, features of irregular shape and heterogeneous density may not be sufficiently characterized by the pair correlation analysis, which summarizes an entire image into a few parameters.

The optimized clustering algorithm we described in this work complements these methods by generating a full superresolution image and accurate list of molecule positions that provide additional qualitative and quantitative information that cannot be described by one or a few global parameters. We have described a few quantitative analyses that can be performed (dimension, mean density, relative density, and density distribution analysis), but each biological system may be characterized by unique quantitative features that can be measured from the images and list of positions our method provides. Additionally, because our method is a simple modification to the superresolution image analysis algorithm first developed for PALM [1], it can be conveniently implemented in commonly used algorithms.

## Experimental Methods and Simulations

### Simulation of FtsZ structures

Based on our experimental PALM images [7], we simulated FtsZ molecules in the cell as two populations: midplane and cytoplasmic. Positions for cytoplasmic molecules were sampled from a uniform distribution across the entire cell area (1 μm ×3 μm rectangle). Positions for midplane molecules were sampled from a uniform distribution in a 110 nm ×1 μm rectangle at the midcell. Figure S8A shows a representative simulation of distributed midplane (red) and cytoplasmic (blue) molecules.

Simulations presented in this work were comprised of 1000, 2000, or 5000, total molecules that were distributed with 30%, 50%, or 75% in the midplane population (see Table S1).

### Simulation of circular clusters

Each simulation of circular clusters required designation of the following parameters: total number of molecules in a cell, fraction of molecules within clusters, mean number of molecules per cluster, and cluster FWHM (parameter ranges can be found in Table S2). The positions of molecules that were not within clusters (cytoplasmic molecules) were sampled in the same manner as described for the cytoplasmic molecules in the FtsZ simulation.

The integer number of clusters in a given simulation was calculated by dividing the total number of molecules in all clusters by the mean number of molecules per cluster. Then, the number of molecules in each cluster was sampled from an exponential distribution. The molecules within each cluster were scattered according to a symmetrical, two-dimensional Gaussian distribution with the designated FWHM, and the center of each cluster was distributed uniformly throughout the cell. Figure S8C shows a representative simulation of distributed clustered (red) and cytoplasmic (blue) molecules.

### Simulation of fluorophore blinking kinetics

We simulated the kinetics of fluorophore activation and blinking by varying the following parameters:

1. Activation rate (*k_1_*): rate at which new molecules are activated; inversely proportional to the number of unactivated molecules remaining.
2. Blink off rate (*k_2_*): rate at which a molecule is reversibly converted to the transient ‘off’ state.
3. Blink on rate (*k_3_*): rate at which a molecule is turned on from the transient ‘off’ state.
4. Bleaching rate (*k_4_*): rate at which a molecule is irreversibly turned off by photobleaching.

Figure 1A shows a diagram of this simple kinetic scheme. Experimentally, *k_1_* is controlled by the intensity of the activation laser and *k_4_* is controlled by the intensity of the excitation laser. The kinetic parameters were simulated using MATLAB® via Monte-Carlo simulations of fluorophore dynamics where exponential probability distributions were assigned for each of the following parameters (expected values were designated at the beginning of the simulations and can be found in Tables S1 and S2):

1. Lag time between molecule activations (*τ_act_*) was sampled from an exponential probability distribution with expected value of *τ_act_^0^(N_initial_/N_remaining_)*, which increases as the number of remaining molecules (*N_remaining_*) that have not been activated decreases. *τ_act_^0^* is the expected lag time at the initial number of molecules (*N_initial_*).
2. The duration of each blink (*τ_on_*).
3. The duration of dark times between blinks (*τ_off_*).
4. Number of blinks per molecule (*n_blink_*).

The *N_initial_* values used in the simulation are listed in Tables S1 and S2. All times were rounded to the nearest integer number of frames (presented figures plot time in seconds using a 50 ms exposure time). Note that the kinetic parameters used in these simulations reflect observed fluorescence time traces that are highly dependent on experimental conditions such as excitation/activation light intensity, pixel size, background noise, frame rate, spot detection algorithm, and spot fitting algorithm. We validated this scheme by verifying that the observed time traces of individual mEos2 molecules under our experimental conditions can be well-described by exponential distributions of *τ_on_*, *τ_off_*, and *n_blink_* (Figure S7A, B, C).

Molecules were iteratively activated with lag times sampled from the updated *τ_act_* distribution. Once activated, the number of blinks for a given molecule was sampled from the *n_blink_* distribution. For each blink event, the on-time was sampled from the *τ_on_* distribution and the time until the next blink was sampled from the *τ_off_* distribution. The lag time (*τ_act_*) until the next molecule activation was calculated from the start of the previously activated molecule such that blinking events from multiple molecules could overlap in time.

For low expected values, sampling from an exponential distribution often resulted in values less than one frame. A value of *n_blink_* <0.5 (rounded to *n_blink_*  = 0) means that the molecule was activated but never ‘observed’ in the simulation due to fast photobleaching. This was also true for low values of mean on-time (*τ_on_*), which resulted in some blinks not being recorded. These properties mimic experimental conditions and highlight the fact that not every activated fluorophore or blinking event will be visualized.

Once the blinking time trace was established for each molecule, its multiple localizations were scattered around the original molecule position according to a two-dimensional normal distribution with a standard deviation, *σ*, between 7–37 nm (see Table S1 and S2). For the simulation described in Figures 2, 3, 4, 5, *σ* = 15 nm, which results in a FWHM spatial resolution of 35 nm. We validated that the position distributions generated by the simulation were similar to those observed for experimental mEos2 localizations by plotting the histograms of distances between repeat localizations of the same molecule that result from the simulation (Figure S7E) and from experimental data (Figure S7D). The shape and peak position of both distributions are similar, suggesting that the simulation with *σ* = 15 nm approximates our experimental data.

### Superresolution image reconstruction with localization-based PSFs

Superresolution images of both simulated and experimental data were generated in MATLAB® with a pixel size of 15 nm. Each localized spot was plotted as a symmetrical 2D Gaussian distribution with total intensity equal to 1 and *σ* equal to the experimental or simulated localization precision. For simulated data, the localization precision was equivalent to the standard deviation, *σ*, used to simulate the scatter of localizations around the molecule position (see previous paragraph). For experimental data, only molecules with localization precisions smaller than 20 nm were plotted. Superresolution images were pseudocolored using the ‘Red Hot’ lookup table in ImageJ software (NIH).

### Superresolution molecule density measurement

For density measurements, superresolution molecule density images of both simulated and experimental data were generated in MATLAB® with a pixel size of 15 nm. The intensity of each pixel in the density image represents the number of molecules detected within the pixel's boundaries, and hence represents a two-dimensional histogram of molecule counts. For experimental data, only molecules with localization precisions smaller than 20 nm were included.

Boundaries of the Z-ring or cluster regions of each density image were identified as polygons by eye. Relative density within the Z-ring (*f_midcell_*) was calculated by dividing the summed intensity within the Z-ring region by the total intensity of the density image (total number of localized spots). Similarly, relative density within clusters (*f_cluster_*) was calculated by dividing the summed intensity within all clusters by the total intensity of the density image. Z-ring density histograms were generated by binning pixels within the Z-ring region by intensity.

### Calculation of maximal activation rate

The average time between molecule activations, *Δt_max_*, was calculated from the time intervals between localizations within the 225 nm×225 nm square of maximum density, which was identified by applying a mean filter to the superresolution density image (see above) using the ‘nlfilter’ function in MATLAB with a 17pixel×17pixel sliding window (15 nm pixel size). The maximum density square is centered at the maximum intensity pixel in the filtered image. This calculation is identical for both simulated and experimental data.

### Clustering algorithm

Both simulated and experimental time traces were clustered in MATLAB® using two thresholds: any spot that occurred within time, *t_Thresh_*, and distance, *d_Thresh_*, of a previous spot was grouped into the same cluster as that previous spot (see Figure S1 for flowchart). The resulting cluster was assigned a spatial position equal to the centroid position of all the spots included in the cluster.

### Jaccard index of cluster accuracy

We calculated the Jaccard index [38], [39] of clustering accuracy for each clustering result as *TP/(TP +FP + FN). TP* is the number of pairs of localizations that came from the same molecule, and were grouped into the same cluster; *FP* is the number of pairs of localizations that did not come from the same molecule, but were grouped into the same cluster; and *FN* is the number of pairs of localizations that came from the same molecule, but were grouped into different clusters. The Jaccard index varies from 0 to 1, where a value of 1 represents perfect clustering.

### Preparation and purification of mEos2

The *mEos2* gene was amplified using primers AATTGTCGACAATGAGTGCGATTAAGCCAGACA and TTAAGCGGCCGCTTATCGTCTGGCATTGTCAG. The PCR product was restricted using *Sal*I and *Not*I restriction enzymes (New England Biolabs), and cloned into the same sites of plasmid pT7HMT [15]. The inserted sequence was confirmed via sequencing and the resulting plasmid was transformed into BL21-Gold(DE3) cells (Stratagene). To purify his-mEos2, cells were cultured overnight at 37°C in LB (Luria Broth) media. The culture was then diluted 1∶200 in 200 mL LB media, grown at 37°C to an OD_600_ of 0.8, then induced for 2 hours with 0.5 mM IPTG (Isopropyl β-D-1-thiogalactopyranoside). Cells were collected at 4100 rpm for 20 min in a Sorvall Legend RT bench top centrifuge at 4°C. Cells were lysed using sonication and repeated freeze thaw cycles and subsequently purified on Ni-NTA beads (Invitrogen) at 4°C according to the ProBond™ native protocol. Protein was buffer-exchanged and concentrated from elution buffer to pH 7.4 phosphate buffer saline using a Vivaspin 500 concentrator (GE Healthcare) with a 5,000 MW cutoff. Purified his-mEos2 was checked for purity by running Mini-PROTEAN TGX Gels (BIO-RAD), and concentration was determined in a Nanodrop spectrophotometer using absorption coefficient of 5,600 M^−1^cm^−1^ at 280 nm [29]. His-tagged mEos2 was diluted 1∶10,000 from a stock concentration of 13.5 µM, then frozen in an ethanol dry-ice bath and stored at −80°C in 2 µL aliquots.

### *In vitro* sample preparation

A 12% NiCl**_2_** aqueous solution was prepared and filtered to sterilize and remove aggregates. A coverslip was secured via Scotch tape (3 M) to a Mini Mouse II (Denville scientific) centrifuge with the 1.5 mL tube attachment. While spinning at 6000 RPM, 50 µL of the NiCl**_2_** solution was slowly pipetted to the center of the spinning coverslip through a ∼1 cm hole in the plastic cover. The spin coating was visually inspected for evenness. Next, 0.5 µL of purified his-mEos2 sample (1.35 nM) was pipetted to the center of the NiCl**_2_**-coated coverslip without spinning. Sample was allowed to dry, then topped with an agarose gel pad to mimic the PALM image acquisition setup described previously [7].

### *E. coli* sample preparation and fixation

*E. coli* B/rA cells containing plasmid pCA24N-FtsZ-mEos2 [7] were grown in M9 media supplemented with glucose at 25°C to an OD_600_ of 0.2, then induced with 20 µM IPTG for 2 hrs. Cells were then collected via centrifugation, resuspended in fresh M9 media, grown at 25°C for 90 minutes, and fixed with 4% formaldehyde at 25°C for 40 min. The fixed *E. coli* cells were mixed with 50 nm gold fiducial beads (Microspheres-Nanospheres, Mahopac, NY), then sandwiched between an agarose gel pad and a cleaned coverslip as described previously [7].

### PALM Imaging conditions

Images were acquired using an Olympus IX-71 inverted microscope, equipped with a 60×, 1.45 NA TIRFM objective. A 405 nm laser (CUBE^TM^, Coherent, Santa Clara, CA) was used to activate mEos2. The green and red fluorescence of mEos2 was excited via epi-illumination with 488 nm and 561 nm solid state lasers (Sapphire, Coherent, Santa Clara, CA) and emission was collected on an EMCCD camera (iXon DU897E, Andor Technology, Belfast, Northern Ireland) after passing through a dual-band emission filter (510/19 and 620/20, Chroma Technology, Rockingham, VT).

During the imaging sequence, the sample was illuminated continuously with both 405 nm and 561 nm lasers. The exposure time was 50 ms per frame (20 Hz) with a 100pixel ×100pixel imaging area. The intensity of the 405 nm activation laser was increased stepwise as the number of unactivated mEos2 molecules decreased during data acquisition.

### Molecule detection in experimental data

Molecules were detected and localized as described previously [7]. Briefly, intensity and area thresholds were used to identify potential fluorescence spots, which were fit to a symmetric, two-dimensional Gaussian function using a nonlinear least squares algorithm in MATLAB. Localization precision was calculated from the photon counts according to the theoretical formula [4]. Sample drift was calibrated by applying the frame-to-frame displacement of 50 nm gold beads, which were localized in the same manner as single molecules of mEos2.

## Supporting Information

Figure S1 **Schematic of spot clustering algorithm.** After spot identification and localization, the set of spots is processed iteratively such that any previous spot (“forerunner spot”) that occurred within *t_Thresh_* and *d_Thresh_* of a given spot (“spot *i*”) are grouped together. Each group is then plotted only once in the final superresolution image at the calculated centroid position.
(TIF)

Figure S2 **Characterization of** ***Δt_repeat_*** **for mEos2.** Histogram of time (in frames) between sequential molecule localizations, *Δt_repeat_*, from 1743 molecules resulting in 3815 localized spots and 2072 intervals between sequential localizations of the same molecules. Data from both *in vitro* samples and fixed cells expressing low levels of mEos2 were combined to generate this histogram. Most repeat localizations occur in consecutive frames (first bin, *Δt_repeat_*  = 1), but long dark intervals (>10 frames) are sometimes observed. The sample mean is <*Δt_repeat_*>  = 2.1±3.3 frames (0.11±0.17 seconds).
(TIF)

Figure S3 **Examples of intersection identification in the |(N – Nref)/Nref| plot.** Among the 92 simulations investigated, three categories of plot shapes were observed: symmetric (A), asymmetric (B), and diffuse (C). The optimal threshold pair identified by eye for each example is shown as a blue circle. For symmetric plots, the optimal threshold pair should be selected in the center of the intersection point. For asymmetric plots, the optimal threshold pair should be selected at the inflection point with the longest *t_Thresh_* value. The identification of the intersection point in diffuse plots (C) may be difficult because the intersection area is broad. However, these plots result from kinetic parameters that yield very broad and high Jaccard peaks such that a broad range of thresholds around the intersection points yield almost equivalent and sufficient accuracy in resulting images. These representative plots were generated from simulated datasets with the following parameters (*N_total_*-midplane %-<*n_blink_*>,<*τ_off_*>,<*τ_on_*>,<*τ^0^_act_*>): 500–30%–1,4,1,1 (A), 1000–30% −2,8,1,1 (B), 500–50% –3,4,1,20 (C) – all *τ* values are reported in frames; 1 frame  = 50 ms).
(TIF)

Figure S4 **Characterization of mEos2 in fixed E. coli cells.** (A) Representative images from a single *E. coli* cell expressing mEos2. The brightfield (top left) and green fluorescence (bottom left) images are shown for comparison. The scatter plot (right) shows single molecule localizations (small dots) colored by detection time. Localizations that originated from the same molecule are grouped together (large circles). The cell outline is shown in white. (B) Histogram (gray) of localizations per mEos2 molecule (α) in fixed *E. coli* cells with the corresponding single-exponential fit (red). The fitted mean is 0.5±0.1 localizations per molecule. The ensemble average is 2.1±4.3 (std. dev., *N* = 1228) localizations per molecule. (C) Histogram (gray) and single-exponential fit (red) calculated after combining *in vitro* (B) and *in vivo* (Figure 8B) datasets, which yielded similar values. The fitted mean is 0.6±0.1 localizations per molecule. The ensemble average is 2.2±3.9 (std. dev., *N* = 1743) localizations per molecule.
(TIF)

Figure S5 **Optimal tThresh values are related to the mean off time.** Datasets from both Z-ring and cluster simulations were grouped by simulated fluorophore off-time, *τ_off_*, then *t_Thresh_* values at the Jaccard index peak of each simulation were averaged (blue circles; error bars represent standard deviation) and plotted against the fluorophore off-time. The two parameters show a clear correlation, suggesting that the optimum *t_Thresh_* value is largely determined by the mean fluorophore off time (linear fit: Y = 3.3X+0.14, R^2^ = 0.99). However, the large variation at some *τ_off_* values suggest that other experimental factors affect the optimal *t_Thresh_* value. A list of simulations used in this analysis can be found in Table S1 and S2.
(TIF)

Figure S6 **Optimal dThresh values are related to the spatial resolution.** Datasets from Z-ring and cluster simulations were grouped by simulated spatial resolution (FWHM), then the *d_Thresh_* values at the Jaccard index peak of each simulation were averaged (blue circles; error bars represent standard deviation) and plotted against the simulated spatial resolution. Spatial resolution was calculated as 2.35*σ*, where *σ* is the Gaussian standard deviation used to scatter localizations around the central molecule positions (see Methods and Text S1). The two parameters show a clear correlation, indicating that larger spatial resolutions result in larger values for optimum d_Thresh_ (linear fit: Y = 1.4X+10.2, R2 = 0.96). This plot was generated using the same datasets analyzed in Figure S5 (see Table S1 and S2 for parameter list).
(TIF)

Figure S7 **Validation of kinetic and spatial simulation parameters.** Combined datasets from both *in vitro* and *in vivo* characterizations of mEos2 were used to generate histograms of *n_blink_* (A), *τ_off_* (B), and *τ_on_*(C), which are all described well by single-exponential distributions, validating the simple kinetic scheme used to simulate fluorophore dynamics in this work. Red curves indicate single-exponential fits that yielded mean values of: <*n_blink_*>  = 0.44±0.03 (A), <τ_off_>  = 2.0±0.2 frames (B), and <*τ_on_*>  = 0.56±0.03 frames (C). (D–E) Histograms of the pair-wise distances between repeat localizations of the same molecule for experimental (D) and simulated (E) datasets. The histograms were fit to Equation 2 from Text S1 (p(Δr)  =  (r/2σ2)*exp(−r2/4σ2); red lines), yielding standard deviation, *σ*, of 18±1 nm (D) and 15±1 nm (E), respectively. The experimental dataset is the same combined dataset characterized in (A–C) above. The simulated dataset had a nominal σ of 15 nm, and is the same dataset analyzed in Figures 2–5 (*N_total_*  = 2000 (50% midplane), *σ* = 15 nm, <*n_blink_*>  = 2, <*τ_off_*>  = 1 frame, <*τ_on_*>  = 1 frame, <*τ^0^_act_*>  = 5 frames (1 frame  = 50 ms)).
(TIF)

Figure S8 **Representative simulations of superresolution images.** (A) Representative simulation of FtsZ locations within an *E. coli* cell. The total number of molecules, *N*, is 500. Molecules are divided into 50% midplane (red) and 50% cytoplasmic (blue). (B) Superresolution image generated from the data in (A) after simulating the following kinetic parameters: <*n_blink_*>  = 3, <*τ_off_*>  = 4 frames, <*τ_on_*>  = 1 frame, <*τ^0^_act_*>  = 7 frames, and applying the same clustering algorithm used to generate Figure 1B: spots within 167 nm (1 camera pixel) and 50 ms (1 frame) of each other were grouped together and plotted once. The simulated image reproduces the time-correlated clusters observed in the experimental image (Figure 1B). (C) Representative simulation of clusters within an *E. coli* cell. The total number of molecules, *N*, is 2000. Molecules are divided into 50% clustered (red) and 50% cytoplasmic (blue) molecules, with the cluster diameter designated as 50 nm FWHM. The number of molecules per cluster was sampled from an exponential distribution with <molecules/cluster>  = 200. (D) Superresolution image generated from the data in (C) after simulating the following kinetic parameters: <*n_blink_*>  = 2, <*τ_off_*>  = 1 frames, <*τ_on_*>  = 1 frame, <*τ^0^_act_*>  = 5 frames, but plotting only the first localization of each molecule. Scale bars, 500 nm. Grid size, 30 nm.
(TIF)

Table S1 **Simulation parameters for Z-ring simulations.**
(XLSX)

Table S2 **Simulation parameters for cluster simulations.**
(XLSX)

Text S1 **Calculation of spatial resolution.**
(DOCX)
