# Supplementary figures and images for: Accurate Construction of Photoactivated Localization Microscopy (PALM) Images for Quantitative Measurements
Source: PLoS One. 2012 Dec 12;7(12):e51725. doi: 10.1371/journal.pone.0051725 (PMC3520911; doi:10.1371/journal.pone.0051725)

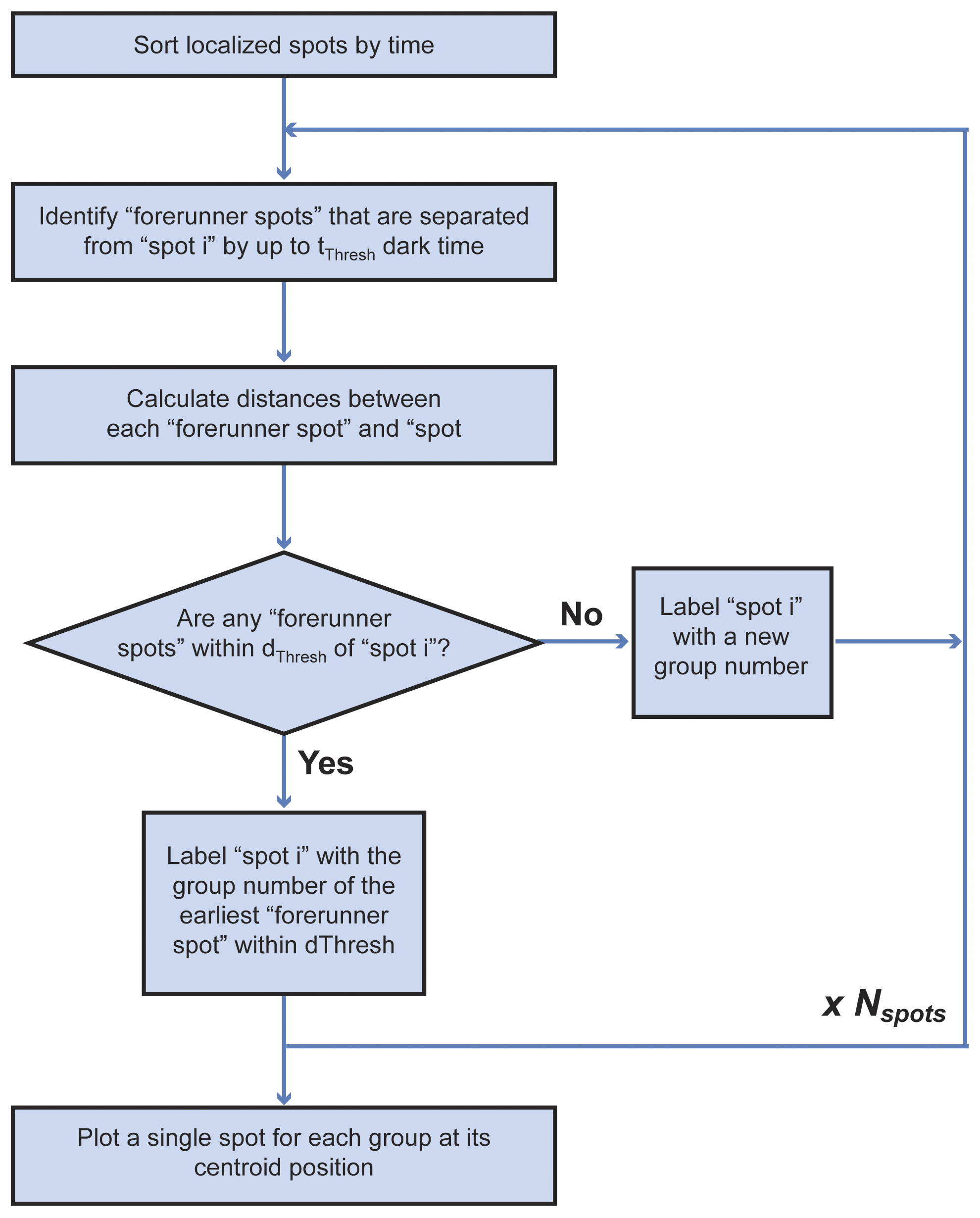

Supplement: Figure S1 — Schematic of spot clustering algorithm. After spot identification and localization, the set of spots is processed iteratively such that any previous spot (“forerunner spot”) that occurred within tThresh and dThresh of a given spot (“spot i”) are grouped together. Each group is then plotted only once in the final superresolution image at the calculated centroid position. (TIF) [file pone.0051725.s001.tif]

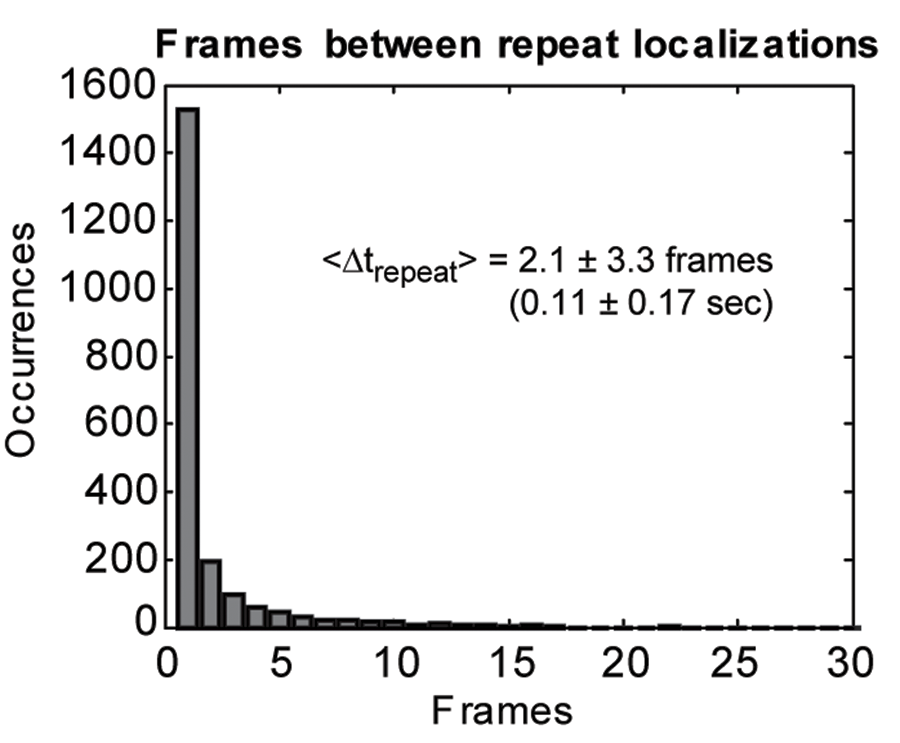

Supplement: Figure S2 — Characterization of Δtrepeat for mEos2. Histogram of time (in frames) between sequential molecule localizations, Δtrepeat, from 1743 molecules resulting in 3815 localized spots and 2072 intervals between sequential localizations of the same molecules. Data from both in vitro samples and fixed cells expressing low levels of mEos2 were combined to generate this histogram. Most repeat localizations occur in consecutive frames (first bin, Δtrepeat = 1), but long dark intervals (>10 frames) are sometimes observed. The sample mean is <Δtrepeat> = 2.1±3.3 frames (0.11±0.17 seconds). (TIF) [file pone.0051725.s002.tif]

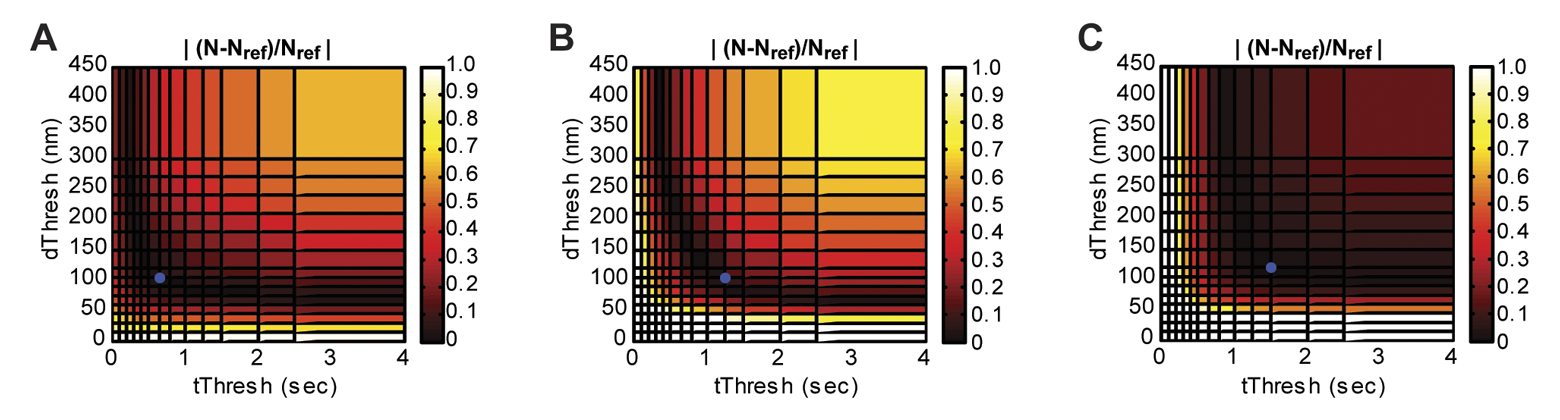

Supplement: Figure S3 — Examples of intersection identification in the |(N – Nref)/Nref| plot. Among the 92 simulations investigated, three categories of plot shapes were observed: symmetric (A), asymmetric (B), and diffuse (C). The optimal threshold pair identified by eye for each example is shown as a blue circle. For symmetric plots, the optimal threshold pair should be selected in the center of the intersection point. For asymmetric plots, the optimal threshold pair should be selected at the inflection point with the longest tThresh value. The identification of the intersection point in diffuse plots (C) may be difficult because the intersection area is broad. However, these plots result from kinetic parameters that yield very broad and high Jaccard peaks such that a broad range of thresholds around the intersection points yield almost equivalent and sufficient accuracy in resulting images. These representative plots were generated from simulated datasets with the following parameters (Ntotal-midplane %-,<τoff>,<τon>,<τ0act>): 500–30%–1,4,1,1 (A), 1000–30% −2,8,1,1 (B), 500–50% –3,4,1,20 (C) – all τ values are reported in frames; 1 frame = 50 ms). (TIF) [file pone.0051725.s003.tif]

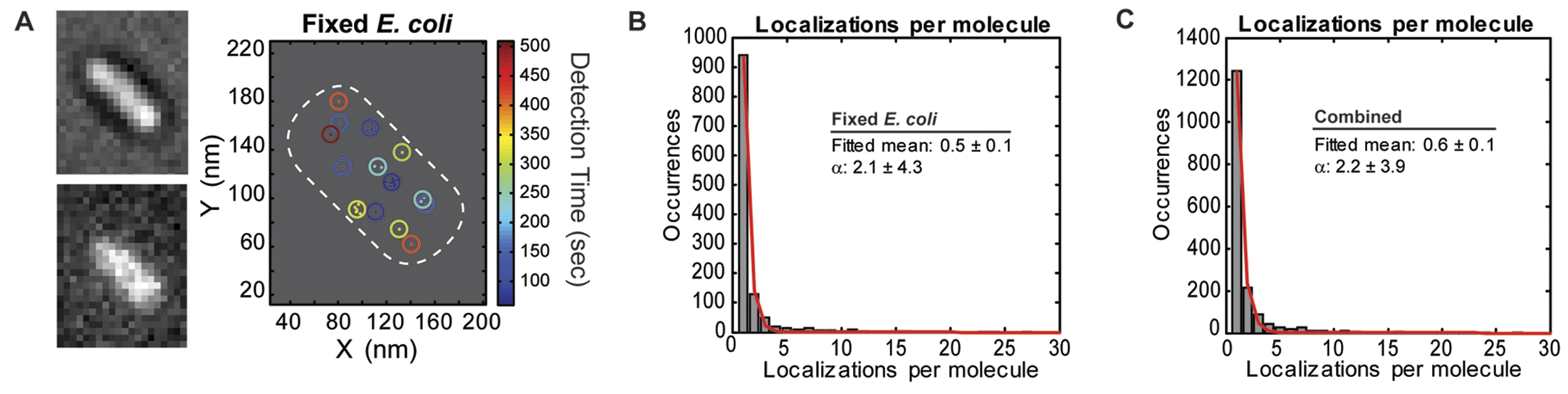

Supplement: Figure S4 — Characterization of mEos2 in fixed E. coli cells. (A) Representative images from a single E. coli cell expressing mEos2. The brightfield (top left) and green fluorescence (bottom left) images are shown for comparison. The scatter plot (right) shows single molecule localizations (small dots) colored by detection time. Localizations that originated from the same molecule are grouped together (large circles). The cell outline is shown in white. (B) Histogram (gray) of localizations per mEos2 molecule (α) in fixed E. coli cells with the corresponding single-exponential fit (red). The fitted mean is 0.5±0.1 localizations per molecule. The ensemble average is 2.1±4.3 (std. dev., N = 1228) localizations per molecule. (C) Histogram (gray) and single-exponential fit (red) calculated after combining in vitro (B) and in vivo (Figure 8B) datasets, which yielded similar values. The fitted mean is 0.6±0.1 localizations per molecule. The ensemble average is 2.2±3.9 (std. dev., N = 1743) localizations per molecule. (TIF) [file pone.0051725.s004.tif]

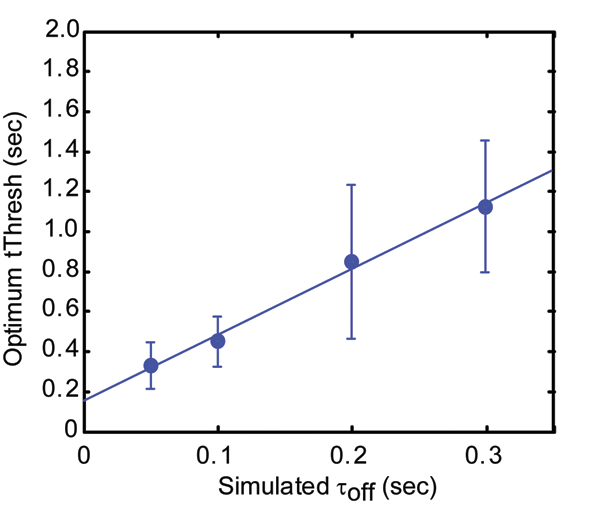

Supplement: Figure S5 — Optimal tThresh values are related to the mean off time. Datasets from both Z-ring and cluster simulations were grouped by simulated fluorophore off-time, τoff, then tThresh values at the Jaccard index peak of each simulation were averaged (blue circles; error bars represent standard deviation) and plotted against the fluorophore off-time. The two parameters show a clear correlation, suggesting that the optimum tThresh value is largely determined by the mean fluorophore off time (linear fit: Y = 3.3X+0.14, R2 = 0.99). However, the large variation at some τoff values suggest that other experimental factors affect the optimal tThresh value. A list of simulations used in this analysis can be found in Table S1 and S2. (TIF) [file pone.0051725.s005.tif]

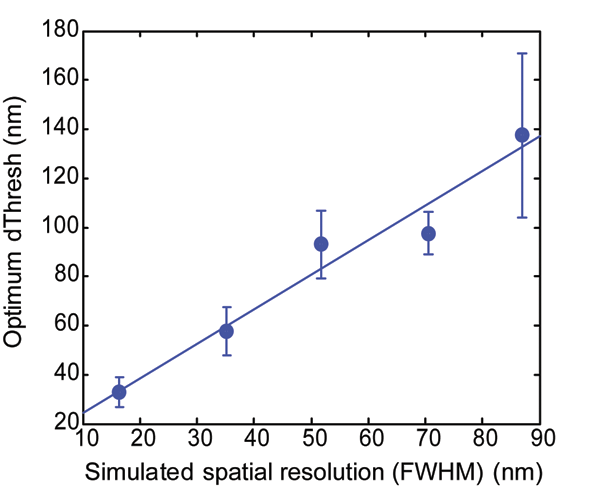

Supplement: Figure S6 — Optimal dThresh values are related to the spatial resolution. Datasets from Z-ring and cluster simulations were grouped by simulated spatial resolution (FWHM), then the dThresh values at the Jaccard index peak of each simulation were averaged (blue circles; error bars represent standard deviation) and plotted against the simulated spatial resolution. Spatial resolution was calculated as 2.35σ, where σ is the Gaussian standard deviation used to scatter localizations around the central molecule positions (see Methods and Text S1). The two parameters show a clear correlation, indicating that larger spatial resolutions result in larger values for optimum dThresh (linear fit: Y = 1.4X+10.2, R2 = 0.96). This plot was generated using the same datasets analyzed in Figure S5 (see Table S1 and S2 for parameter list). (TIF) [file pone.0051725.s006.tif]

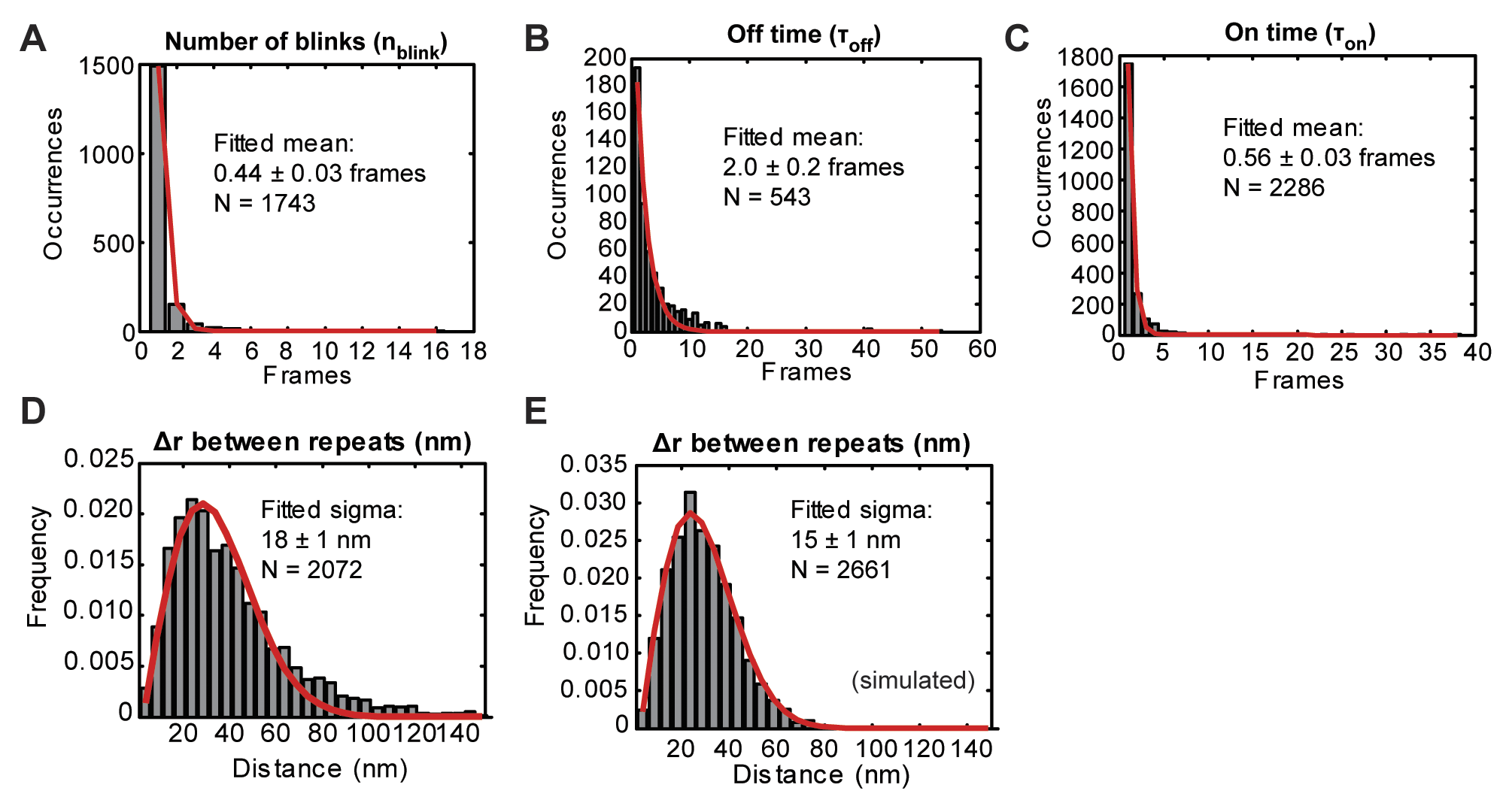

Supplement: Figure S7 — Validation of kinetic and spatial simulation parameters. Combined datasets from both in vitro and in vivo characterizations of mEos2 were used to generate histograms of nblink (A), τoff (B), and τon(C), which are all described well by single-exponential distributions, validating the simple kinetic scheme used to simulate fluorophore dynamics in this work. Red curves indicate single-exponential fits that yielded mean values of: = 0.44±0.03 (A), <τoff> = 2.0±0.2 frames (B), and <τon> = 0.56±0.03 frames (C). (D–E) Histograms of the pair-wise distances between repeat localizations of the same molecule for experimental (D) and simulated (E) datasets. The histograms were fit to Equation 2 from Text S1 (p(Δr) = (r/2σ2)*exp(−r2/4σ2); red lines), yielding standard deviation, σ, of 18±1 nm (D) and 15±1 nm (E), respectively. The experimental dataset is the same combined dataset characterized in (A–C) above. The simulated dataset had a nominal σ of 15 nm, and is the same dataset analyzed in Figures 2–5 (Ntotal = 2000 (50% midplane), σ = 15 nm, = 2, <τoff> = 1 frame, <τon> = 1 frame, <τ0act> = 5 frames (1 frame = 50 ms)). (TIF) [file pone.0051725.s007.tif]

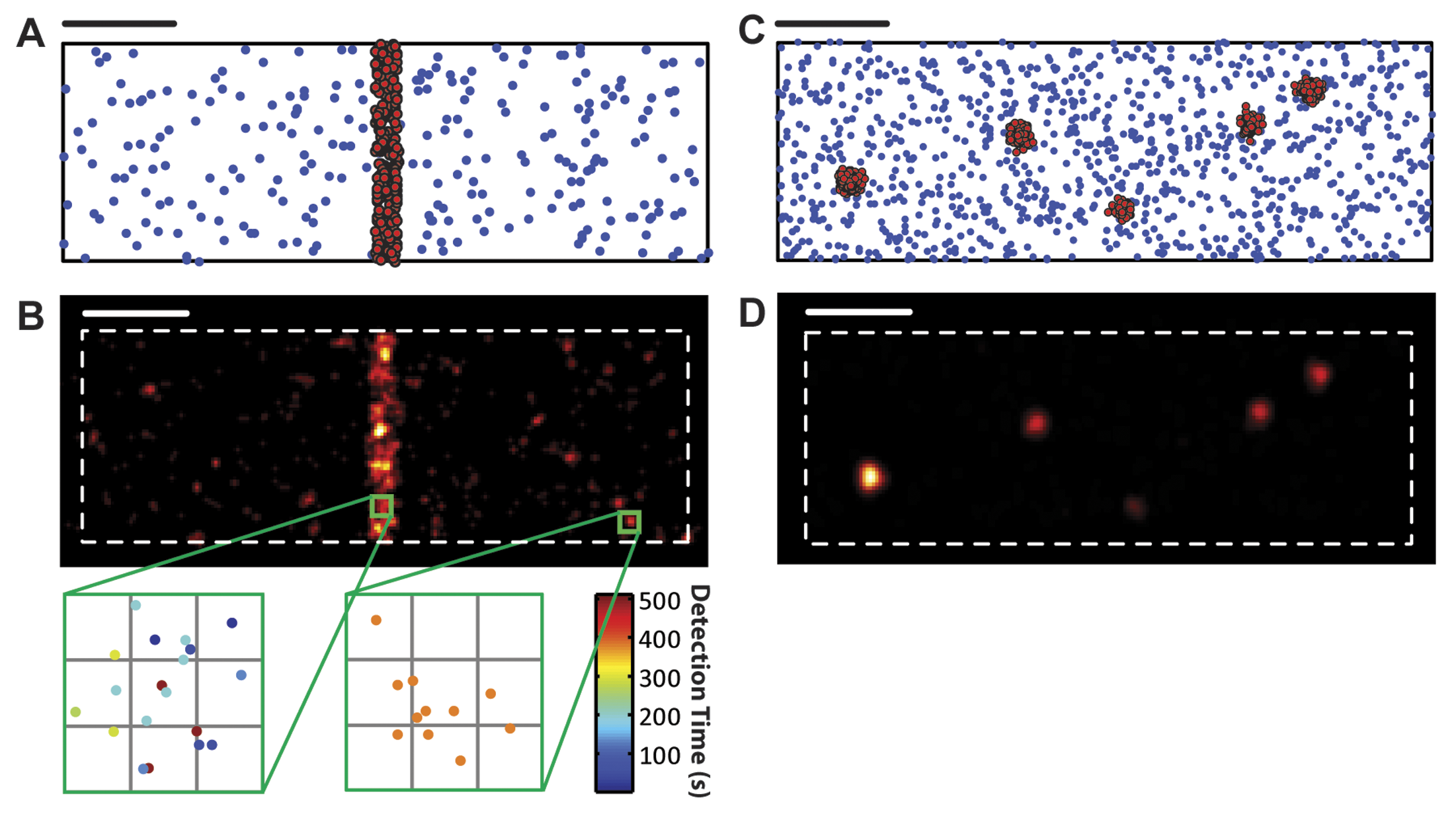

Supplement: Figure S8 — Representative simulations of superresolution images. (A) Representative simulation of FtsZ locations within an E. coli cell. The total number of molecules, N, is 500. Molecules are divided into 50% midplane (red) and 50% cytoplasmic (blue). (B) Superresolution image generated from the data in (A) after simulating the following kinetic parameters: = 3, <τoff> = 4 frames, <τon> = 1 frame, <τ0act> = 7 frames, and applying the same clustering algorithm used to generate Figure 1B: spots within 167 nm (1 camera pixel) and 50 ms (1 frame) of each other were grouped together and plotted once. The simulated image reproduces the time-correlated clusters observed in the experimental image (Figure 1B). (C) Representative simulation of clusters within an E. coli cell. The total number of molecules, N, is 2000. Molecules are divided into 50% clustered (red) and 50% cytoplasmic (blue) molecules, with the cluster diameter designated as 50 nm FWHM. The number of molecules per cluster was sampled from an exponential distribution with <molecules/cluster> = 200. (D) Superresolution image generated from the data in (C) after simulating the following kinetic parameters: = 2, <τoff> = 1 frames, <τon> = 1 frame, <τ0act> = 5 frames, but plotting only the first localization of each molecule. Scale bars, 500 nm. Grid size, 30 nm. (TIF) [file pone.0051725.s008.tif]
