# Supplementary material for: Accurate Construction of Photoactivated Localization Microscopy (PALM) Images for Quantitative Measurements
Source: PLoS One. 2012 Dec 12;7(12):e51725. doi: 10.1371/journal.pone.0051725 (PMC3520911; doi:10.1371/journal.pone.0051725)
Supplement: Text S1 — Calculation of spatial resolution. (DOCX) [file pone.0051725.s011.docx]

**Text S1. Calculation of spatial resolution**

The spatial resolution of PALM or STORM experiments is determined by several factors. The theoretical upper bound of spatial resolution is related to the localization precision (*σ*), which primarily depends on the number of photons detected per localized spot [[1](#_ENREF_1)]:

$\sigma^{2}= \frac{s^{2}+{a^{2}}/{12}}{N}+\frac{8\pi s^{4}b^{2}}{a^{2}N^{2}}$ (1)

where *s* is the standard deviation of the Gaussian point spread function, *α* is the image pixel size, *N* is the number of photons detected from the molecule, and *b* is the background noise. The upper bound of spatial resolution is then the FWHM (full width at half maximum) of a Gaussian distribution with localization precision, *σ,* as the standard deviation*.*

In practice, the experimental spatial resolution is often defined as the positional variability in repeat localizations of the same molecule, which can deviate from the theoretical localization precision value due to thermal fluctuations and nonidealities in the detection system. For molecules that are localized many times (such as STORM dyes), this positional variability can be measured by first identifying the mean position of each molecule, then superimposing localizations of many molecules such that the mean positions of every molecule are aligned. The FWHM of the resulting Gaussian distribution of positions is the maximum achievable spatial resolution[[2](#_ENREF_2)], given sufficient sampling (see below). When each molecule is only localized a few times (as with PALM fluorophores), however, the mean position of each molecule is less well-defined and this method is not as reliable.

For PALM fluorophores, measurement of positional variability can be made from the distribution of pair-wise displacement between repeat localizations of the same molecule. This measurement is more applicable for fluorophores that are only localized a few times because the results from many molecules can be combined into a single distribution without alignment. The distribution of distances between two localized spots is given by the following equation [[3](#_ENREF_3)]:

$p\left( r \right)=\left( \frac{r}{\sigma_{1}^{2}+\sigma_{2}^{2}} \right){e^{\left( -\frac{\mu^{2}+r^{2}}{2\left( \sigma_{1}^{2}+\sigma_{2}^{2} \right)} \right)}I}_{0}\left( \frac{r\mu}{\sigma_{1}^{2}+\sigma_{2}^{2}} \right)$ (2)

where *p(r)* is probability of observing displacement, *r*, in two dimensions; *σ_1_* is the standard deviation in localized positions for spot 1; *σ_2_* is standard deviation in localized positions for spot 2; *μ* is the true distance between the two spots; and *I_0_* is the modified Bessel function of integer order zero. For calculation of spatial resolution, the true distance between repeat localizations of the same molecule is zero (*μ* = 0) and the standard deviations in localized positions for both spots are identical (*σ_1_ = σ_2_ = σ*), so Equation 2 simplifies to:

$p\left( r \right)=\left( \frac{r}{2\sigma^{2}}e^{\frac{-r^{2}}{4\sigma^{2}}} \right)$ (3)

where *p(r)* is probability of observing displacement, *r*, between repeat localizations.­­­ The achieved spatial resolution is then the FWHM calculated from the measured *σ* (FWHM *= 2.35σ*). Equation 3 was used to fit the distribution of distances between repeat localizations for both experimental (Figure S7D) and simulated (Figure S7E) datasets to extract the spatial resolution. For the simulated dataset, the fitted spatial resolution (*σ* = 15 ± 1nm; FWHM = 35 ± 2nm) was in excellent agreement with the nominal spatial resolution (*σ* = 15nm; FWHM = 35nm).

The measurements of spatial resolution provided above all reflect the precision of identifying a single molecule's location. It is important to note that the spatial resolution needed to resolve a given structural feature is also related to the sampling of the structure by the Nyquist-Shannon criterion, which states that a structure needs to be sampled at greater than twice the desired resolution[[4-6](#_ENREF_4)]. For example, to achieve a precision-dictated FWHM resolution of 35nm, a structure needs to be sampled once every 17.5 nm on average. Thus, a 350x350 nm^2^ structure would require 400 molecules to achieve 35nm resolution, and detection of only 100 molecules would yield 70nm resolution.

**References**

1. Thompson RE, Larson DR, Webb WW (2002) Precise Nanometer Localization Analysis for Individual Fluorescent Probes. Biophysical Journal 82: 2775-2783.

2. Bates M, Huang B, Dempsey GT, Zhuang X (2007) Multicolor Super-Resolution Imaging with Photo-Switchable Fluorescent Probes. Science 317: 1749-1753.

3. Stirling Churchman L, Flyvbjerg H, Spudich JA (2006) A Non-Gaussian Distribution Quantifies Distances Measured with Fluorescence Localization Techniques. Biophysical Journal 90: 668-671.

4. Shroff H, Galbraith CG, Galbraith JA, Betzig E (2008) Live-cell photoactivated localization microscopy of nanoscale adhesion dynamics. Nat Meth 5: 417-423.

5. Nyquist H (1928) Certain topics in telegraph transmission theory. Trans AIEE 47: 617-644.

6. Shannon CE (1949) Communication in the presence of noise. Proc Institute of Radio Engineers 37: 10-21.
